# Construction of an immune-related signature with prognostic value for colon cancer

**DOI:** 10.7717/peerj.10812

**Published:** 2021-05-05

**Authors:** Yunxia Lv, Xinyi Wang, Yu Ren, Xiaorui Fu, Taiyuan Li, Qunguang Jiang

**Affiliations:** 1 Department of Thyroid Surgery, The Second Affiliated Hospital of Nanchang University, Nanchang, Jiangxi, China; 2 Queen Mary College, Medical Department, Nanchang University, Nanchang, Jiangxi, China; 3 Department of First Clinical Medical College, Nanchang University, Nanchang, China; 4 Department of Gastrointestinal Surgery, The First Affiliated Hospital of Nanchang University, Nanchang, Jiangxi, China

**Keywords:** Colon cancer, Immune related gene, Signature, Tumor immune microenvironment, Nomogram

## Abstract

**Background:**

Colon cancer is the third most common malignant tumor in the world. Although immunotherapy has been used in cancer treatment, there is still no first-line immunotherapy method for colon cancer. Therefore, it is essential to search for potential immunotherapy targets and molecular biomarkers for early diagnosis and prognosis.

**Methods:**

In this study, we downloaded transcriptome data from The Cancer Genome Atlas (TCGA) and immune-related genes from the ImmPort database. Then we filtered genes with prognostic value and constructed an immune-related signature. Patients were classified into low- and high-risk groups, and we exerted a series of analysis between the signature and clinical phenotypes. Additionally, we used protein-protein interaction networks, gene set enrichment analysis (GSEA) and single-sample gene-set enrichment analysis (ssGSEA) to explore the underlying mechanism of this signature. Furthermore, the accuracy of this signature was verified, using two data sets from Gene Expression Omnibus (GEO).

**Results:**

We selected 12 immune-related genes to construct the immune-related signature. Low-risk group had a higher level of immunity compared to high-risk group. The expression level of HLA genes and checkpoint-related genes were statistically different in low- and high-risk groups. This signature showed its prognostic value in TCGA cohort and 2 GEO data sets. The signature also had strong correlation with TNM classification, stage, survival state and lymphatic invasion. The mechanism of the signature may be related to several transcription factors and CD8+ T cell in the tumor microenvironment.

**Conclusion:**

In conclusion, this immune-related signature is of great prognosis value for colon cancer and its biofunction might be correlated with HLA genes, checkpoint-related genes and high-infiltrating T cells in tumor tissues.

## Introduction

Colon cancer ranks the third most common malignant tumor and fourth leading cause of cancer-related death, with over 1 million new cases are diagnosed worldwide every year (International Agency for Research on Cancer (IARC), 2019). The five-year survival rate of patients with stage I colon cancer is around 90%, while less than 10% in patients with stage IV (Labianca et al., 2013). According to the report of the Objective National Central Cancer Registry of China, although the treatment based on surgery and chemotherapy have made great progress, the prognosis of patients with colon cancer is still poor as most of them are diagnosed when the tumor has already entered the advanced stage (Chen et al., 2017). Therefore, it is particularly essential to study the pathogenesis of colon cancer and search for molecular biomarkers which can provide suggestion for early diagnosis and prognosis.

The etiology of colon cancer is not very clear. Dietary habits, environmental pollution, intestinal flora, gene mutations and family inheritance have been proved to be related with the occurrence of colon cancer. It has been reported that either polyposis or nonpolyposis syndromes can contribute to the genetic vulnerability to colon cancer, which is correlated with deletion or mutation in FAP (APC gene) and several DNA mismatch repair genes (Rustgi, 2007; Kwong & Dove, 2009; Church, 2016). Additionally, microsatellite instability (MSI), KRAS and BRAF mutational status have also been considered as prognostic factors for colon cancers and may give clues for adjuvant therapy in the future (Boland & Goel, 2010; Arrington et al., 2012; Prahallad et al., 2012; Cullis, Das & Bar-Sagi, 2018). In the last decade, immune checkpoint-inhibiting agents, such as programmed death-1 receptor (PD1) and cytotoxic T-lymphocyte antigen 4 (CTLA-4) inhibitors, have been developed as antitumor drugs (Duraiswamy et al., 2013; Yaghoubi et al., 2019). Early trial showed that PD1 and PD-L1 blockade appear to be a promising choice for colon cancer patients with MSI (Binnewies et al., 2018). The viewpoint that the immune system can influence the progression of cancer has been the hotspot for study over a century. Recently, numerous evidences indicated that the tumor immune microenvironment (TIM) was of great value in predicting prognosis and evaluating therapeutic efficacy factors (Binnewies et al., 2018). TIM is composed of immune cells, immune-related pathways and cytokines that secreted by immune cells (Lazarus et al., 2018). Poor outcomes of patients with colon cancer can be predicted by characteristics of the TIM, such as increased TGF*β* level, low infiltrating level of T cells, suppressed activity of T helper cells (Tauriello et al., 2018). However, there are still several shortcomings which remain to be further studied and improved in immunotherapy for colon cancer. For example, the identification of first-line treatment method for patients with up-regulated or down-regulated expression of specific genes.

Over the past years, the relationship between chronic inflammation and colon cancer had been well demonstrated, but the crucial role of immune cells in tumorigenesis was rarely reported (Terzić et al., 2010). The immune cells infiltrate into tumor tissues and generate inflammatory cytokines, such as IL-6, IL-10 and IL-12 (Ullman & Itzkowitz, 2011). These immune cells can regulate gene expression in tumor cells, so as to contribute to initiation, development and migration of malignant cancer (Gout & Huot, 2008; Gajewski, Schreiber & Fu, 2013). Villarreal et al., (2018) demonstrated that mAb therapy targeting CCR8, a chemokine receptor on T-regs, can significantly suppress tumor growth by enhancing infiltration of CD4+ and CD8+ T cells and improve long-term survival of colorectal tumor. O’Malley et al. (2018) stated that tumor-associated stromal cells can enhance colon tumor progression by supporting PD-L1-inducted T cell suppression. A recent study reported that the activation of STAT3 played a significant role in increasing infiltration of CD8+ lymphocytes and inhibiting the recruitment of T-regs which enhance colon tumor progression and immune escape (Lin et al., 2011). However, there has been no research that can systematically explore the characteristics of TIM in colon cancer and evaluate the correlation between immune-related genes and prognosis of patients. And the regulatory mechanism of immune-related genes also calls for exploration, as to identify new targets for immunotherapies and foster novel immune-based approaches.

In this study, we aimed to construct an immune-related signature for colon cancer based on transcriptome data from The Cancer Genome Atlas (TCGA) database. In order to evaluate the prognostic value of the signature, we estimated the prognosis risks of patients and exerted a series of statistical correlation analysis between the signature and clinical phenotypes. Additionally, we used protein-protein interaction networks GSEA and ssGSEA to explore the underlying regulatory mechanism of this signature, including transcription factors (TFs) and tumor infiltrating immune cells. Furthermore, the accuracy of this signature was verified in the GEO database, which proved our study was of great value in providing clues for early diagnostic biomarkers and immune-therapy targets for colon cancer.

## Materials & Methods

### Data source

The transcriptome data and clinical information of colon cancer patients were obtained from the TCGA database (https://portal.gdc.cancer.gov/repository) and the GEO database (http://www.ncbi.nlm.nih.gov/geo). In order to construct the signature, we downloaded RNA-seq (level 3, HTSeq-FPKM data) of 445 colon cancer patients (445 primary tumor tissue and 41 solid normal tissue) with complete clinical information from the TCGA database. The microarray data of colon cancer patients were downloaded from GSE17536 (*N* = 177) and GSE29621 (*N* = 65) datasets in the GEO database, which were used for verifying the accuracy of the signature. The clinical information of patients from TCGA and GEO databases are summarized in Table 1. The immune-related genes were obtained from the Immport database (http://immport.niaid.nih.gov), containing 2,498 genes in total (Table S1). The gene list of 318 TFs was obtained from the Cistrome database (http://cistrome.org/db/, Table S2). The relative levels of tumor infiltrating immune cells obtained from the TIMER database (https://cistrome.shinyapps.io/timer) were corresponded with every patient sample from TCGA.

**Table 1:** Summarized clinical information of the included datasets.

**Characteristics**		**Total TCGA**	**Train set (*N* = 224)**		**Test set (*N* = 221)**		**GSE17536 (*N* = 177)**		**GSE29621 (*N* = 65)**	
		**N**	**N**	**%**	**N**	**%**	**N**	**%**	**N**	**%**
**Age (years)**	<60	133	63	0.28	70	0.32	59	0.33		
	≥60	312	161	0.72	151	0.68	118	0.67		
**Gender**	Male	212	123	0.55	110	0.50	96	0.54	40	0.62
	Female	233	101	0.45	111	0.50	81	0.46	25	0.38
**T**	T1	10	6	0.03	4	0.02			0	0.00
	T2	76	47	0.21	29	0.13			8	0.12
	T3	302	145	0.65	157	0.71			52	0.80
	T4	56	26	0.12	30	0.14			5	0.08
	unknown	1	0	0.00	1	0.00			0	0.00
**M**	M0	328	171	0.76	157	0.71			46	0.71
	M1	61	25	0.11	36	0.16			18	0.28
	unknown	56	28	0.13	28	0.13			1	0.02
**N**	N0	264	141	0.63	123	0.56			32	0.49
	N1	102	48	0.21	54	0.24			25	0.38
	N2	79	35	0.16	44	0.20			7	0.11
	unknown	0	0	0.00		0.00			1	0.02
**Stage**	Stage I	75	46	0.21	29	0.13	24	0.14	7	0.11
	Stage II	174	88	0.39	86	0.39	57	0.32	22	0.34
	Stage III	124	60	0.27	64	0.29	57	0.32	18	0.28
	Stage IV	61	25	0.11	36	0.16	39	0.22	18	0.28
	unknown	11	5	0.02	6	0.03	0	0.00	0	0.00
**Lymphatic invasion**	No	245	127	0.57	118	0.53				
	Yes	159	74	0.33	85	0.38				
	unknown	41	23	0.10	18	0.08				
**Venous invasion**	No	292	143	0.64	149	0.67				
	Yes	95	49	0.22	46	0.21				
	unknown	58	32	0.14	26	0.12				
**Fustat**	Alive	351	185	0.83	166	0.75	104	0.59	40	0.62
	Dead	94	39	0.17	55	0.25	73	0.41	25	0.38
**Futime**		829.66 ± 759.19	802.27 ± 749.33		857.42 ± 769.76		1443.67 ± 979.35		1376.15 ± 857.32	

### Constructing the immune-related signature of colon cancer

We exerted Wilcoxon signed-ranked tests to screen differentially expressed immune-related genes between normal tissue samples and primary tumor tissue samples from TCGA, with — FC (Fold change) —>1 and false discovery rate (FDR) < 0.05. The patients with colon cancer from TCGA were randomly divided into a training set (*N* = 224) and a testing set (*N* = 221), using a R package called “caret”. For training set, univariate Cox proportional hazard model (CoxPH) was used to screen immune-related genes with prognostic value (*P* < 0.05). The least absolute shrinkage and selection operator (LASSO) regression model (iteration = 1,000) with an elastic-net penalty was performed for further screening, using a R package called “glmnet” (Friedman, Hastie & Tibshirani, 2010). Then multivariate CoxPH was performed to screen out genes which were used to construct the immune-related signature. The signature gave patients in both training and testing set risk scores based on model coefficients of multivariate CoxPH:

\begin{eqnarray*}\text{Risk score}=\sum _{i=0}^{N}(\text{Expi*Coei}). \end{eqnarray*}

Patients were classified into low- and high-risk group based on the median of risk scores.

### Internal and external validation of the signature

The univariate and multivariate analyses were performed for both clinical phenotypes the signature. The Kaplan–Meier (K–M) survival curves and log-rank test were generated to evaluate the difference in survival between high-risk group and low-risk group. We performed receiver operating characteristic (ROC) curves to measure the prognostic capacity of our signature using a R package called “survivalROC” (Heagerty, Lumley & Pepe, 2000). For further validation of this signature, we also generated K-M survival curves of patients from GEO database. Mann-Whitney and Kruskal-Wallis tests were used to verify whether there was statistical difference in risk scores between different clinical subtypes.

### Transcription factors interaction network and gene set enrichment analysis

Using RNA-seq data from TCGA, we screened the differentially expressed TFs between solid normal tissue and primary tumor tissue samples by Wilcoxon signed-ranked tests and calculated the Pearson correlation coefficients between differentially expressed TFs and genes in our immune signature. We visualized the interaction networks of TFs with Cor>0.4 and *P* < 0.05 and genes in the signature using a software called “Cytoscape” (Shannon, 2003). We performed GSEA (Subramanian et al., 2005) to explore gene ontology (GO) terms related to our immune signature. Gene ontology gene sets (c5.bp.v7.0.symbols.gmt) were obtained from Molecular Signatures Database (MSigDB, http://software.broadinstitute.org/gsea/downloads.jsp). When FDR was less than 0.25, the enriched gene set was considered to be statistically significant.

### Analyses of immune signatures enrichment and immune cell infiltrating

In order to analyze related immune pathways and immune cells of our signature, we quantified the enrichment level of immune signatures by ssGSEA using R package “GSVA” (Subramanian et al., 2005), and gene set (Barbie et al., 2009) was used to assess the score of every gene set for every sample. Then, the immune cell infiltration score of primary tumor tissue was quantified by ESTIMATE (Yoshihara et al., 2013). We demonstrated the correlation between risk score and relative immune cell infiltrating level by calculating the Pearson correlation coefficients. The proportions of the 22 tumor infiltrating immune cells were determined by using a R package called “CIBERSORT” (Newman et al., 2015). The tumor purity scores, relative infiltrating immune cells and expression levels of human leukocyte antigen (HLA) were compared between high risk and low risk groups by Mann-Whitney U test.

## Results

### Constructing and verifying an immune-related signature for colon cancer

**Figure 1:**
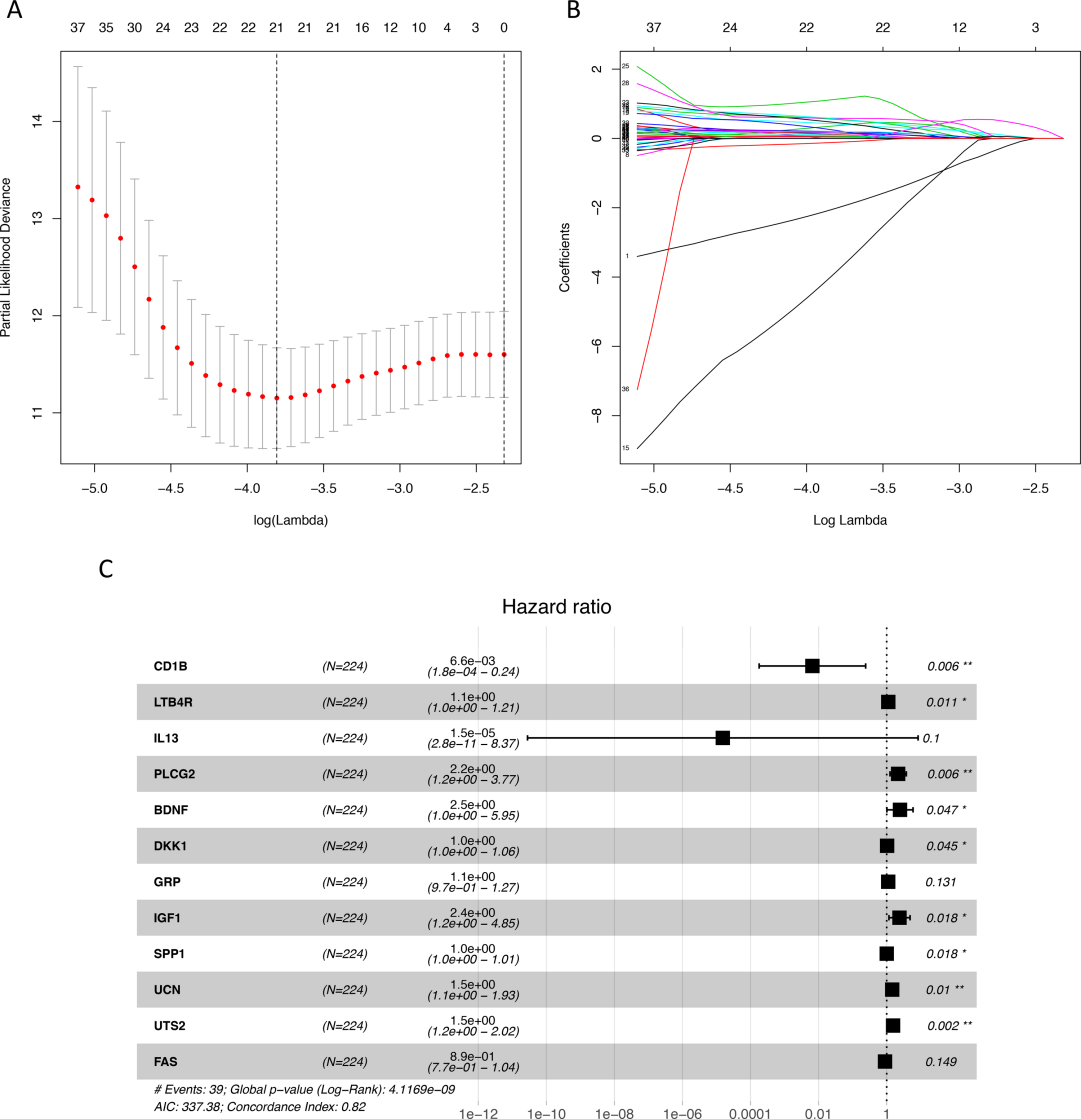
Identification of immune-related genes with prognostic value. (A–B) LASSO analysis identified 21 genes which were most correlated to overall survival in training set. (C) After further screening of immune-related genes by multivariate CoxPH regression model, we obtained 12 genes for construction of immune-related signature. LASSO, least absolute shrinkage and selection operator; CoxPH, Cox proportional hazard model.

We identified 211 down-regulated and 203 up-regulated immune-related genes in tumor samples from TCGA (Fig. S1, Table S3). There were 45 genes with prognostic capacity after the univariate CoxPH (*P* < 0.05) in the training cohort (Table S4). The 45 genes underwent LASSO cox analysis and 21 genes were selected after 1,000 iteration (Figs. 1A and 1B). Then using multivariate CoxPH regression model (Fig. 1C), we chose 12 gene to construct the immune-related signature. Risk score was estimated as follows:

\begin{eqnarray*}\text{Risk score}= \left( -5.026\times \mathrm{CD}1\mathrm{B} \right) + \left( 0.108\times \mathrm{LTB}4\mathrm{R} \right) + \left( -11.087\times \mathrm{IL}13 \right) + \left( 0.775\times \text{PLCG}2 \right) \nonumber\\\displaystyle + \left( 0.898\times \text{BDNF} \right) + \left( 0.027\times \mathrm{DKK}1 \right) + \left( 0.104\times \mathrm{GRP} \right) + \left( 0.865\times \mathrm{IGF}1 \right) + \left( 0.003\times \mathrm{SPP}1 \right) \nonumber\\\displaystyle + \left( 0.372\times \mathrm{UCN} \right) + \left( 0.432\times \mathrm{UTS}2 \right) + \left( -0.113\times \mathrm{FAS} \right) . \end{eqnarray*}

**Figure 2:**
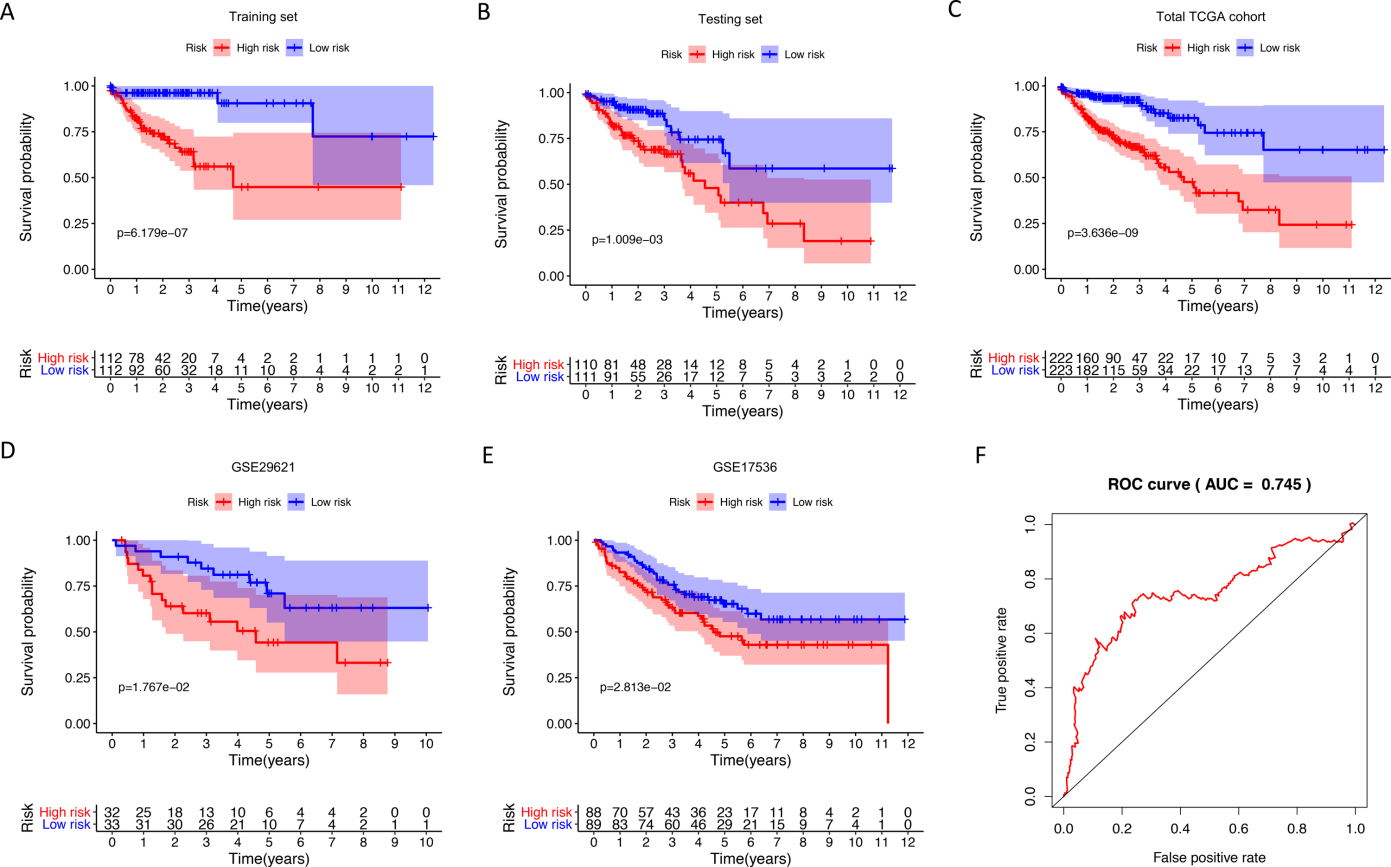
Internal and external validation immune-related signature to determine its clinical prognostic value. (A–E) Kaplan-Meier analyses showed that patients in high-risk group had poorer prognosis compared with low-risk group: training set (*P* = 6.179e−07), testing set (*P* = 1.009e−03), total TCGA colon cancer cohort (*P* = 3.636e−09), GSE29621 (*P* = 1.767e−02), GSE17536 (*P* = 2.813e−02). (F) The AUC of ROC curve was 0.745 in predicting survival time in total TCGA colon cancer cohort. TCGA, The Cancer Genome Atlas; ROC, receiver operating characteristic; AUC, area under the ROC curve.

**Figure 3:**
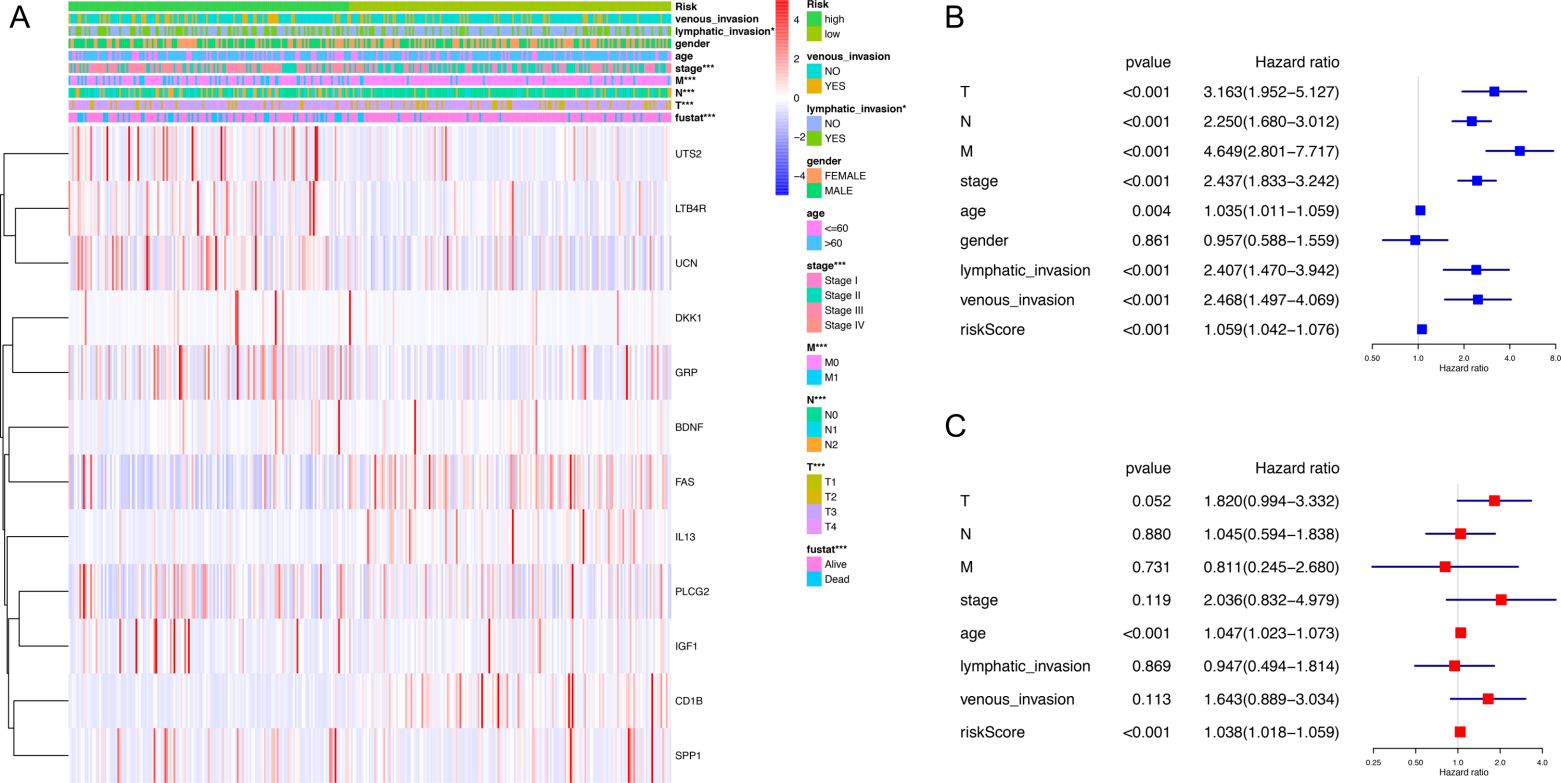
Correlation between clinical phenotypes and expression levels of genes in the signature. (A) We compared differences in venous invasion, lymphatic invasion, gender, age, stage, TNM classification and vital status between low- and high-risk groups by chi-square test (* *P* < 0.05, ** *P* < 0.01, *** *P* < 0.001). The relative expression levels of 12 genes in our immune-related signature were shown in this heatmap. (B) The HR of the signature in univariate analysis cohort was 1.059, with 95% CI from 1.042 to 1.076 (*P* < 0.001). (C) The HR of the signature in multivariate analysis was 1.038, with 95% CI from 1.018 to 1.059 (*P* < 0.001). HR, hazard ratio; CI, confidence interval.

Patients were classified into low- and high-risk groups using the median risk score as the cutoff value. The K-M survival curves were performed to illustrate the difference between the low- and high-risk groups in overall survival: training set (*P* = 6.179e−07, Fig. 2A), testing set (*P* = 1.009e−03, Fig. 2B), total TCGA colon cancer cohort (*P* = 3.636e−09, Fig. 2C), GSE29621 (*P* = 1.767e−02, Fig. 3D), GSE17536 (*P* = 2.813e−02, Fig. 2E). Furthermore, the ROC curve analysis in total TCGA colon cancer cohort, demonstrating promising prognosis value of the signature for colon cancer over survival (AUC = 0.745, Fig. 2F). All of these analyses showed that patients in low-risk group had better prognosis than high-risk group and this immune signature is of strong accuracy in predicting the clinical outcomes of patients with colon cancer.

### The immune-related signature was correlated with clinical phenotypes

**Figure 4:**
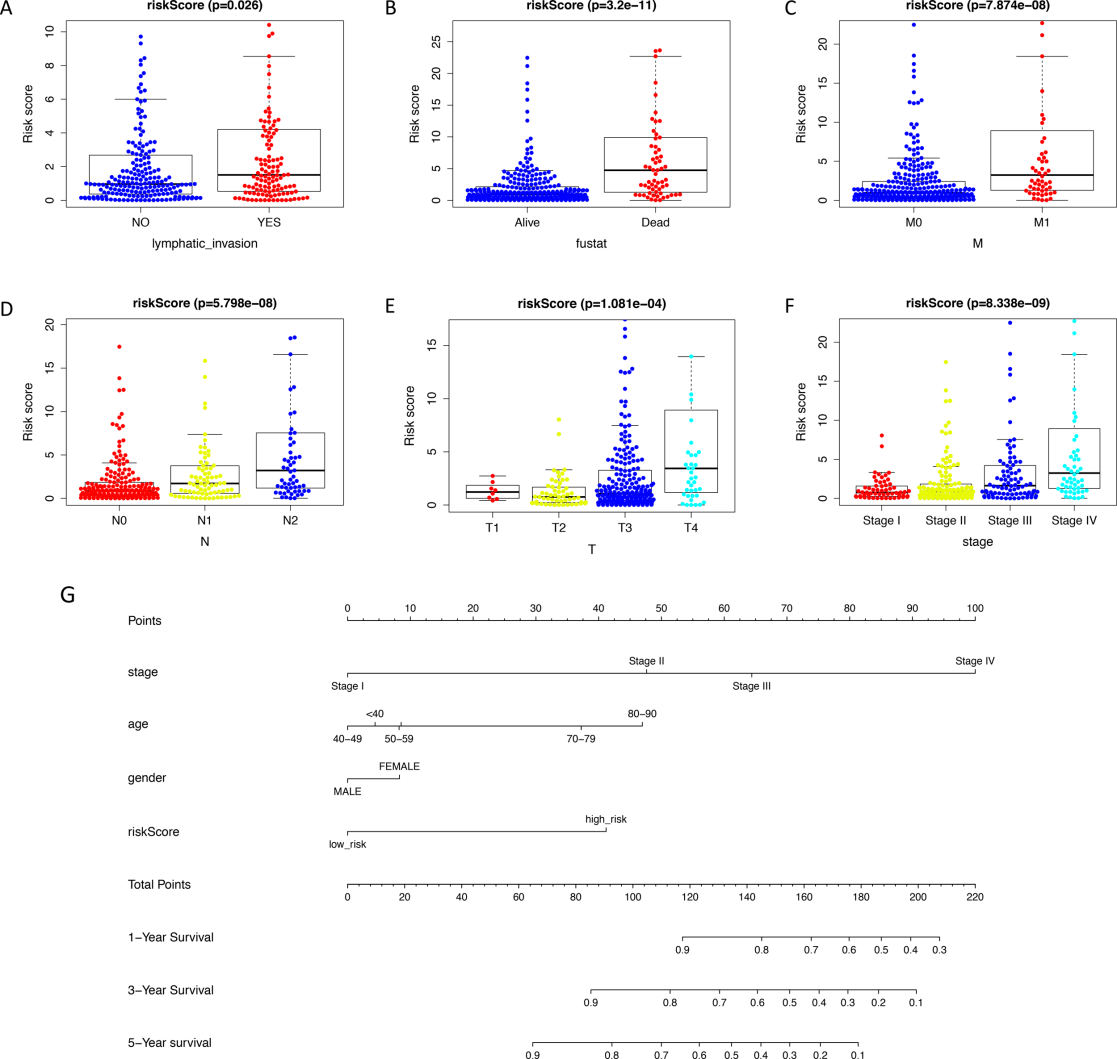
Validating prognostic capacity of the immune signature. (A–F) Using Mann Whitney test and Kruskal-Wallis test, we compared risk scores of samples with different clinical phenotypes (lymphatic invasion, vital status, TNM classifications and stage). This signature was statistically correlated all of these phenotypes. (G) A nomogram for predicting the survival probability of patients with colon cancer with 1-, 3- and 5-year OS. OS, overall survival.

The expression level of immune-related genes and clinical phenotypes of patients in low- and high-risk groups were shown in the heatmap (Fig. 3A). As demonstrated in Fig. 3B, T classification (*P* < 0.001), N classification (*P* < 0.001), M classifications (*P* < 0.001), stage (*P* < 0.001), age (*P* = 0.004), lymphatic invasion (*P* < 0.001), venous invasion (*P* < 0.001) and risk score (*P* < 0.001) are all prognostic factors for colon cancer. In multivariate analysis, risk score was capable to be an independent prognostic factor for colon cancer, with *P* < 0.001, HR = 1.038 and [95%CI] = 1.018–1.059 (Fig. 3C). Additionally, age was also an independent prognostic factor for colon cancer (*P* < 0.001, HR = 1.047, [95%CI] = 1.023–1.073). Then we assessed whether there was statistical difference in clinical phenotypes between low- and high-risk groups by chi-square test. It was indicated that the high-risk group was related to lymphatic invasion, advanced stage, higher level of T, N, M classifications and death in total TCGA colon cancer cohort. Using Mann Whitney test and Kruskal-Wallis test, we compared risk scores of samples with different clinical phenotypes (Figs. 4A–4F), including lymphatic invasion (*P* = 0.026), vital status (*P* = 3.2e−11), M classification (*P* = 7.874e−08), N classification (*P* = 5.798e−08), T classification (*P* = 1.0081e−04) and stage (*P* = 8.338e−09), and found same result with the heatmap. We also compared the expression levels of every gene in this immune-related signature between different clinical subgroups and found most of these genes had strong correlation with stage, T classification and vital status (Table S5). Furthermore, a prognostic nomogram was constructed based on the previous prognostic factors (Fig. 4G).

### Exploration of underlying regulatory mechanism and biological function of the immune-related signature

**Figure 5:**
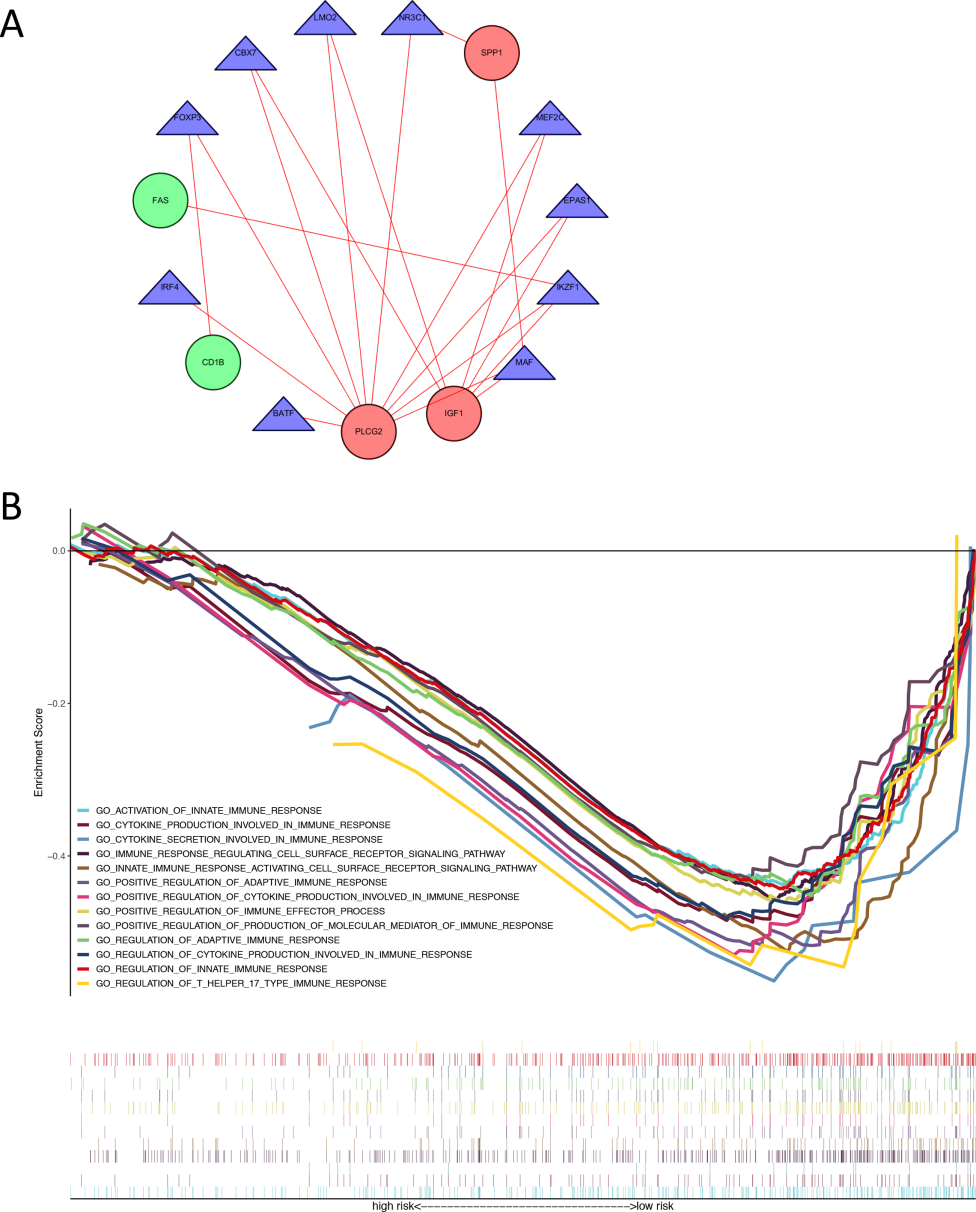
Exploration of underlying regulatory mechanism and biological function of the immune-related signature. (A) The interaction network of genes in the signature and differentially expressed TFs. Red circles (SPP1, PLCG2, IGF1) represent up-regulated immune-related genes. Green circles (CD1B, FAS) represent down-regulated immune-related genes. Blue triangles represent differentially expressed TFs. Red lines mean those two genes have Cor >0.4 and *P* < 0.05 in Person correlation analysis. (B) Gene set enrichment analysis result revealed the significantly enriched immune processes between two risk score levels (high risk and low risk). TF, transcription factor.

We identified 74 differentially expressed TFs between normal and tumor samples from TCGA, with 47 up-regulated and 27 down-regulated TFs (Table S6). We calculated the Pearson correlation coefficients and constructed a protein-protein interaction (PPI) network between differentially expressed TFs and genes in our immune-related risk signature (Fig. 5A). As shown in the interaction network, immune-related gene PLCG2 and IGF1, which are up-regulated in colon tumor samples, have significantly strong correlation with several TFs: FOXP3, IKZF1, CBX7, LMO2, MEF2C, EPAS1 and MAF. These TFs might take part in the regulatory mechanism and biology function of our immune-related signature. The result of GSEA demonstrated that the signature is correlated with some immune pathways (Fig. 5B): positive regulation of adaptive immune response, T-cell-mediated cytotoxicity, secretion of interleukins and cytokines, activation of immune cell surface receptors and T helper 17 type immune response.

### Correlations with risk scores and tumor immune cell infiltrating

**Figure 6:**
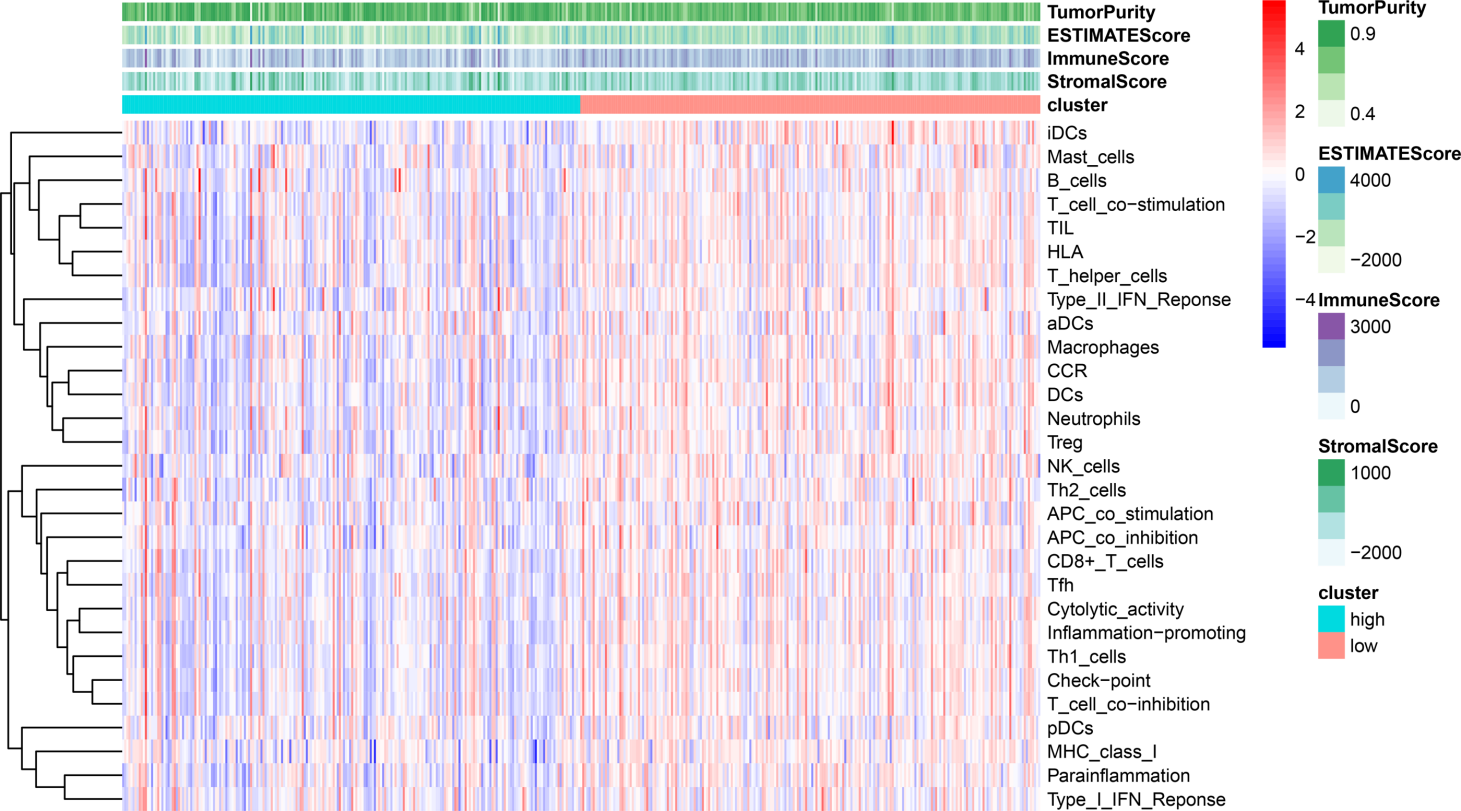
ssGSEA result revealed correlation between risk score and immune-related gene sets. We used ssGSEA to analyze differences in enrichment scores of immune-related gene sets between low- and high-risk groups. These gene sets were composed of immune cells and immune processes. The tumor purity, ESTIMATE score, immune score and stromal score are also shown in this heatmap.

**Figure 7:**
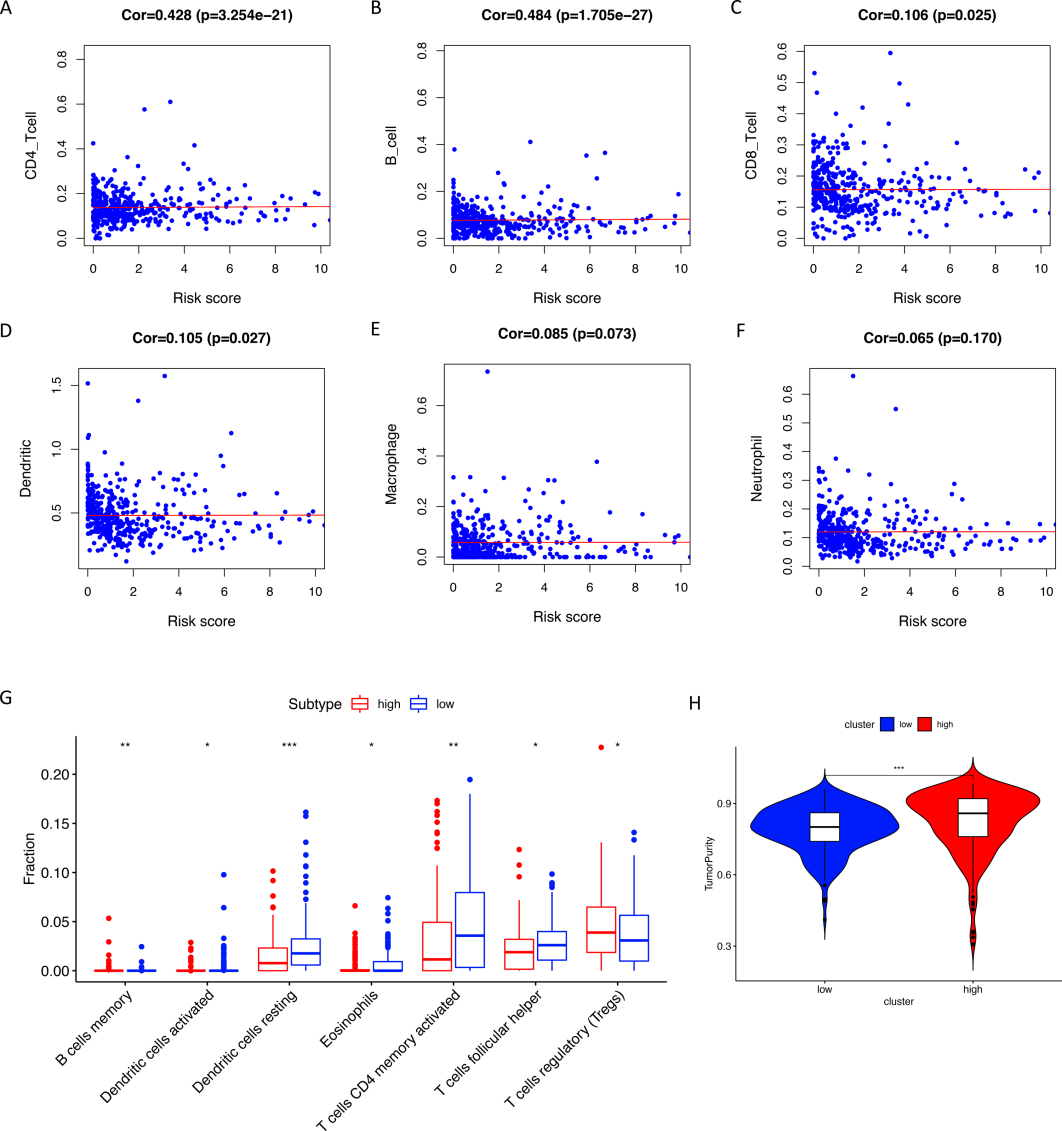
Tumor infiltrating immune cell analysis. (A–F) The Pearson correlation coefficients between immune cell infiltrating levels and risk scores. (G) The relative infiltrating levels of immune cells in two risk groups (* *P* < 0.05, ** *P* < 0.01, *** *P* < 0.001). Two risk groups showed significantly differences in infiltration levels of B cells, dendritic cells, eosinophils, CD4+ T cells, T helper cells and T-regs. (H) The low-risk group showed lower tumor purity than the high-risk group.

**Figure 8:**
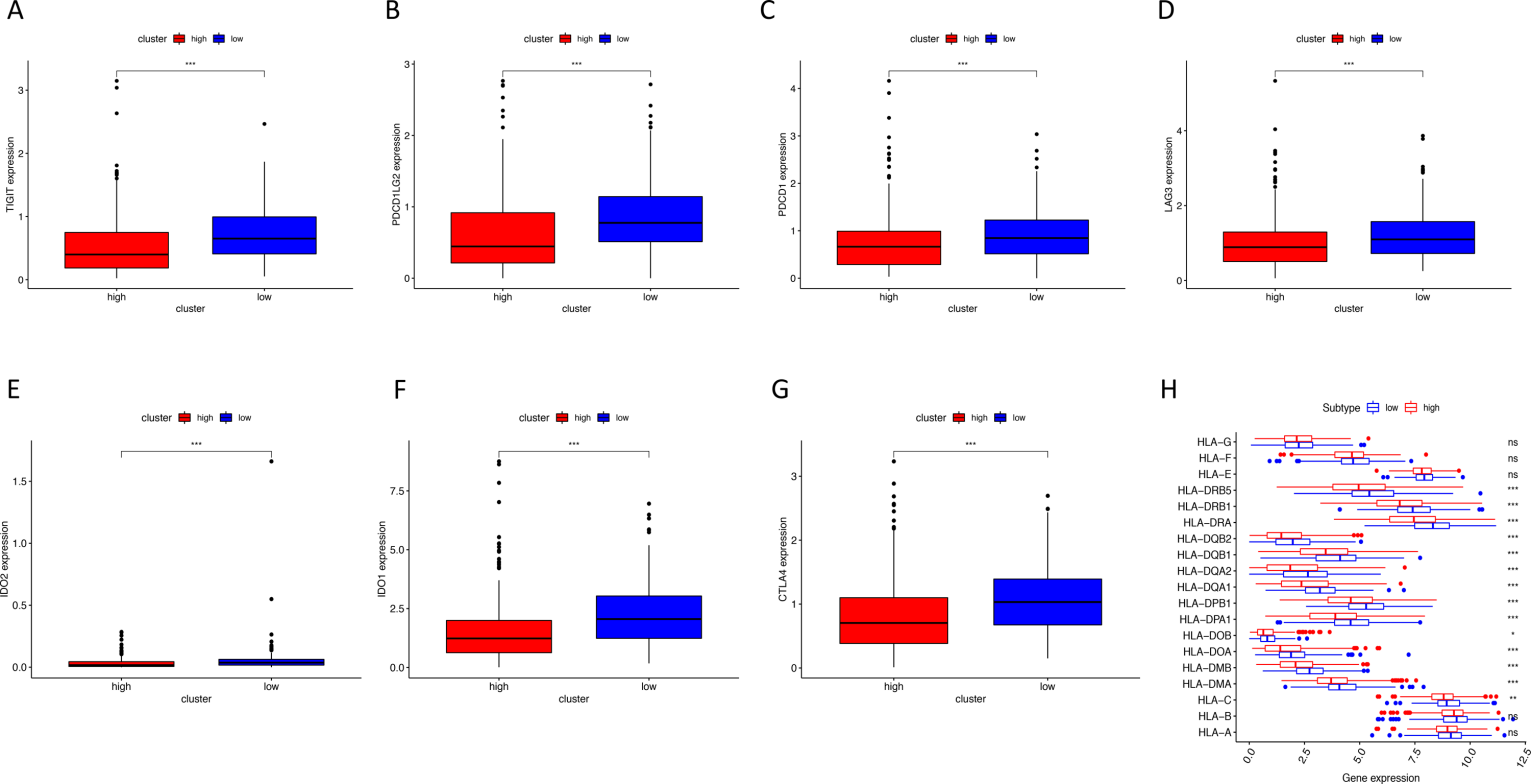
The correlation between risk score and expression levels of HLA and checkpoint-related genes. (A–G) The low-risk group had higher expression levels of seven checkpoint-related genes (TIGIT, PDCD1L, PDCD1, LAG3, IDO2, IDO1 and CTLA4). (H) The low-risk group showed higher expression levels of 14 HLA genes. HLA, human leukocyte antigen. (* *P* < 0.05, ** *P* < 0.01, *** *P* < 0.001).

Using ssGSEA, low-risk group showed higher enrichment level of 29 immune signatures, including inflammation promoting, immune cell stimulation and inhibition, HLA and the relative level of immune cells infiltrating in colon tumor tissues (Fig. 6, Fig. S2). We can notice that the relative infiltrating levels of B cells (Cor = 0.428, *P* = 3.254e−21) and CD4+ T cells (Cor = 0.484, *P* = 1.705e−27) have strong correlation with risk score (Figs. 7A–7F), which meant that immune-related genes in the signature might influence the tumor immune microenvironment by promoting B cells and CD4+ T cells to infiltrate into the tumor tissue. Furthermore, we used “CIBERSORT” to validate relative infiltrating level of 22 types of immune cells (Fig. 7G). It was worth noting that CD4 T cells had higher levels in low-risk group, while T-regs had lower infiltrating level in low-risk group, which meant activity of CD4 T cells are upregulated in the samples with low risk scores. In the ESTIMATE analysis, the tumor purity is lower in low-risk group, which means there are more immune cells infiltrating into tumor tissue. (Fig. 7H). In order to explore the relationship between the signature and immunotherapy, we compared the expression levels of checkpoint-related genes in low- and high-risk groups and found all of them were differentially expressed: TIGIT (*P* < 0.001, Fig. 8A), PDCD1L (*P* < 0.001, Fig. 8B), PDCD1 (*P* < 0.001, Fig. 8C), LAG3 (*P* < 0.001, Fig. 8D), IDO2 (*P* < 0.001, Fig. 8E), IDO1 (*P* < 0.001, Fig. 8F) and CTLA4 (*P* < 0.001, Fig. 8G). Among 19 HLA genes (Fig. 8H), 14 (73.7%) were upregulated in low-risk group, while no HLA genes were upregulated in high-risk groups. As HLA genes encode MHC proteins which take part in the regulation of the immune system, this immune-related risk signature may influence the immune microenvironment of colon cancer cells through regulating HLA genes.

## Discussion

Although colon cancer and rectal cancer were often studied together, more and more histological, genetic and methylation studies supported the idea that rectal carcinoma was different from tumors of the proximal colon (Muzny et al., 2012). As a result, it is of great significance to explore specific biomarkers and prognostic factors for colon cancer, not just generalized standards for colorectal cancer. As colon cancer patients who are diagnosed at early stage have much better prognosis than those who are diagnosed at later stage, it is essential to develop examination method and search for more reliable biomarkers for early diagnosis. In this study, we used transcriptome data and gene-sets obtained from databases to screen out differentially expressed immune-related genes and constructed an immune-related signature. This immune signature was able to predict the survival of colon cancer patients and was significantly correlated with clinical phenotypes, such as venous and lymph node invasion, cancer stage and TNM classification. Importantly, we found this immune signature had strong correlation with immune cell infiltration and was involved in many immune pathways. All of these suggested that this immune signature is of great value for predicting colon cancer patients’ prognosis.

This immune-related risk signature contained 12 genes with prognostic capacity. Most of these genes are cytokines and cytokine receptors, which have been proved to be involved in numerous malignant biological properties of colon cancer, including immune invasion, tumor cell motility, metastasis, recurrence and resistant to immune checkpoint inhibitors. In this signature, up-regulated DKK1 and GRP together with down-regulated FAS and CD1B had significant correlation with tumor stage, metastasis and lymphatic invasion. DKK1, a target of WNT, has been convinced to be a biomarker for predicting colon cancer recurrence and give suggestions for adjuvant therapy stratification in stage II colon cancer (Kandimalla et al., 2017). The over-expression of gastrin releasing peptide (GRP) and its receptor can be discovered after malignant transformation of colon cancer cells, and they can also enhance tumor attachment to the extracellular matrix and promote cytolysis of NK cells (Carroll et al., 2000; Rivera et al., 2009). GRP is regarded as a growth factor of cancer which regulates cancer cell motility by mediating its morphogenic properties (Glover et al., 2005). Fas is a member of the TNFR superfamily, which play an important role in regulating cell growth and intercellular communication in the immune system. Fas signaling pathways is associated with Inflammation activities involving many kinds of immune cells, such as macrophages, epithelial cells and dendritic cells. Fas signaling pathway can also induced the secretion of cytokines and chemokines and transduce powerful inflammatory signals (Cullen & Martin, 2015). CD1B encodes a type of transmembrane glycoproteins, which can sever as both self and foreign lipid antigens to T-cell receptors. Numerous studies showed that CD1B-restricted T cells responded more effectively to lipid from tumor cells than normal cells and leaded to regulation of tumor growth (Chancellor, Gadola & Mansour, 2018). Above all, genes in our immune-related signature have been proved to take part in the progression of colon cancer. However, their relationships with clinical parameters and underlying potential in immunotherapy call for deeper exploration and validation.

Recently, immune cell infiltrating in tumor tissue has become a hotspot in the field of cancer immunotherapy. Inhibition of PD-1-PD-L1 immune checkpoint has showed effectiveness in colon cancer therapy but is still not used as the first-line treatment, as the lack of mechanism explorations and clinical experiments. Our study showed that there were more CD4+ T cell infiltration and higher expression levels of checkpoint-related genes (TIGIT, PDCD1L, PDCD1, LAG3, IDO2, IDO1 and CTLA4) in the low-risk group. However, there are two possible mechanisms which can lead to the upregulation of checkpoint genes: (1) As the infiltration of immune cells also increased in the low-risk group, the upregulation of checkpoint genes may be merely the fellow-up effect of increased immune cell infiltration. (2) The upregulation of checkpoint genes is due to genomic changes in the tumor cell itself, rather than immune cells in the microenvironment of tumor cells. To validate these two possibilities, further cell and animal experiments are needed. CD4+ T cells activate both humoral and cell-mediated responses. Maccalli. C et al found that an antigen of colorectal cancer can elicit an anti-cancer response mediated by CD4+ T cells (Welters et al., 2008). Immune response which involves different T cell population can be activated and integrate anti-tumor activities. Besides, the efficacy of tumor immune surveillance and deregulation of tumor growth depends on the presence of suppressor and regulatory CD4+ T cells in tumor tissue, para-tumor tissue or peripheral blood. As a result, infiltrating T cells may serve as a target for immunotherapy and prognostic factor in colon cancer. Zhao et al. (2017) used CD5-2 to inhibit vascular endothelial-cadherin (VE-cadherin) in tumor-related blood vessels and observed an increase in CD8+ T cells infiltrating and cytotoxicity, which gave another idea of using T-cell infiltrating in tumor immunotherapy. As shown in ssGSEA and HLA analysis, inflammation, activation and inhibition of immune response and expression of HLA genes may also provide possible molecular mechanism or applicable direction for our immune-related risk signature.

For further study, we will not only focus on immune characteristics of colon cancer, but also the regulatory mechanism and biological function of immune-related signature. We identified 74 differentially expressed TFs between normal and tumor samples from TCGA, calculated the Pearson correlation coefficients and constructed a protein-protein interaction (PPI) network between differentially expressed TFs and genes in our immune-related risk signature. Among those TFs, FOXP3 has been widely studied. An essential part of Treg cell function is the expression of FOXP3. Deficiency in FXOP3 can lead to immune dysregulation and plays a role in the occurrence of endocrinopathy and enteropathy. Its function in immune regulation may depend on TGF-*β* signaling pathway and signaling through the T cell receptor, co-receptors and TGF-*β*RI or TGF-*β*RII receptors combine to promote the differentiation of Treg cells (Kerdiles et al., 2010). Besides, our signature should be used to clinic to assess its prognostic capacity, and genes in this signature also need deeper mechanism exploration for their potential clinical application.

## Conclusions

We filtered 12 immune-related genes (CD1B, LTB4R, IL13, PLCG2, BDNF, DKK1, GRP, IGF1, SPP1, UCN, UTS2 and FAS) with prognostic value for colon cancer and used them to construct an immune-related signature. Based on validation in the testing set, TCGA colon cancer cohort and two GEO data sets, we can conclude that this signature was of significant value for predicting the survival and progression (TNM classification, stage and lymphatic invasion) of patient with colon cancer. The risk score generated by this signature was statistically correlated with the expression of HLA genes, checkpoint-related genes, adaptive immunity and infiltration of T cells and dendritic cells. Six TFs (IKZF1, CBX7, LMO2, MEF2C, EPAS1 and MAF) may play roles in the regulatory mechanism of genes in the signature.

##  Supplemental Information

10.7717/peerj.10812/supp-1 Figure S1 Expression levels of immune-related genes in solid normal samples and primary tumor samples

10.7717/peerj.10812/supp-2 Figure S2 Comparison of ssGSEA score between low- and high-risk groups Low-risk group showed significantly higher enrichment level of 29 known immune signatures than high-risk group (Mann-Whitney U test, *P* < 0.05).

10.7717/peerj.10812/supp-3 Table S1 The comprehensive list of 2498 immune-related genes obtained from the Immport database

10.7717/peerj.10812/supp-4 Table S2 The gene list of 318 TFs obtained from the Cistrome database

10.7717/peerj.10812/supp-5 Table S3 Screening of differentially expressed immune-related genes We exerted Wilcoxon signed-ranked tests to screen immune-related genes (—FC (Fold change) —> 1, *P* < 0.05 and FDR < 0.25) between normal tissue samples and primary tumor tissue samples from TCGA. FDR, false discovery rate; FC, fold change; TCGA, The Cancer Genome Atlas.

10.7717/peerj.10812/supp-6 Table S4 Further screening of immune-related genes We used univariate CoxPH to screen out genes with prognosis capacity (*P* < 0.05). There were 45 genes selected. CoxPH, Cox proportional hazard model.

10.7717/peerj.10812/supp-7 Table S5 Validation of correlation between risk scores and clinical phenotypes Using Mann Whitney U test and Kruskal-Wallis test, we compared expression levels of every gene in this immune-related signature in different clinical subgroups. If *P* < 0.05, this gene was considered to be related to this clinical phenotype. As shown in this table, CD1B, PLCG2, DKK1 and GRP had strong correlation with tumor stage, TNM classification or vital status of patients.

10.7717/peerj.10812/supp-8 Table S6 Screening of differentially expressed TFs We exerted Wilcoxon signed-ranked tests to screen differentially expressed transcription factors (—FC (Fold change) —> 1, *P* < 0.05 and FDR < 0.25) between normal tissue samples and primary tumor tissue samples from TCGA. FDR, false discovery rate; FC, fold change; TCGA, The Cancer Genome Atlas.

## Additional Information and Declarations

### Data Availability

The following information was supplied regarding data availability: The RNA-seq transcriptome data and corresponding clinicopathological information were obtained from TCGA: TCGA-COAD. The validation cohort data is also available at NCBI GEO: GSE17536, GSE29621.
