# Supplementary figures and images for: Construction of an immune-related signature with prognostic value for colon cancer
Source: PeerJ. 2021 May 5;9:e10812. doi: 10.7717/peerj.10812 (PMC8106397; doi:10.7717/peerj.10812)

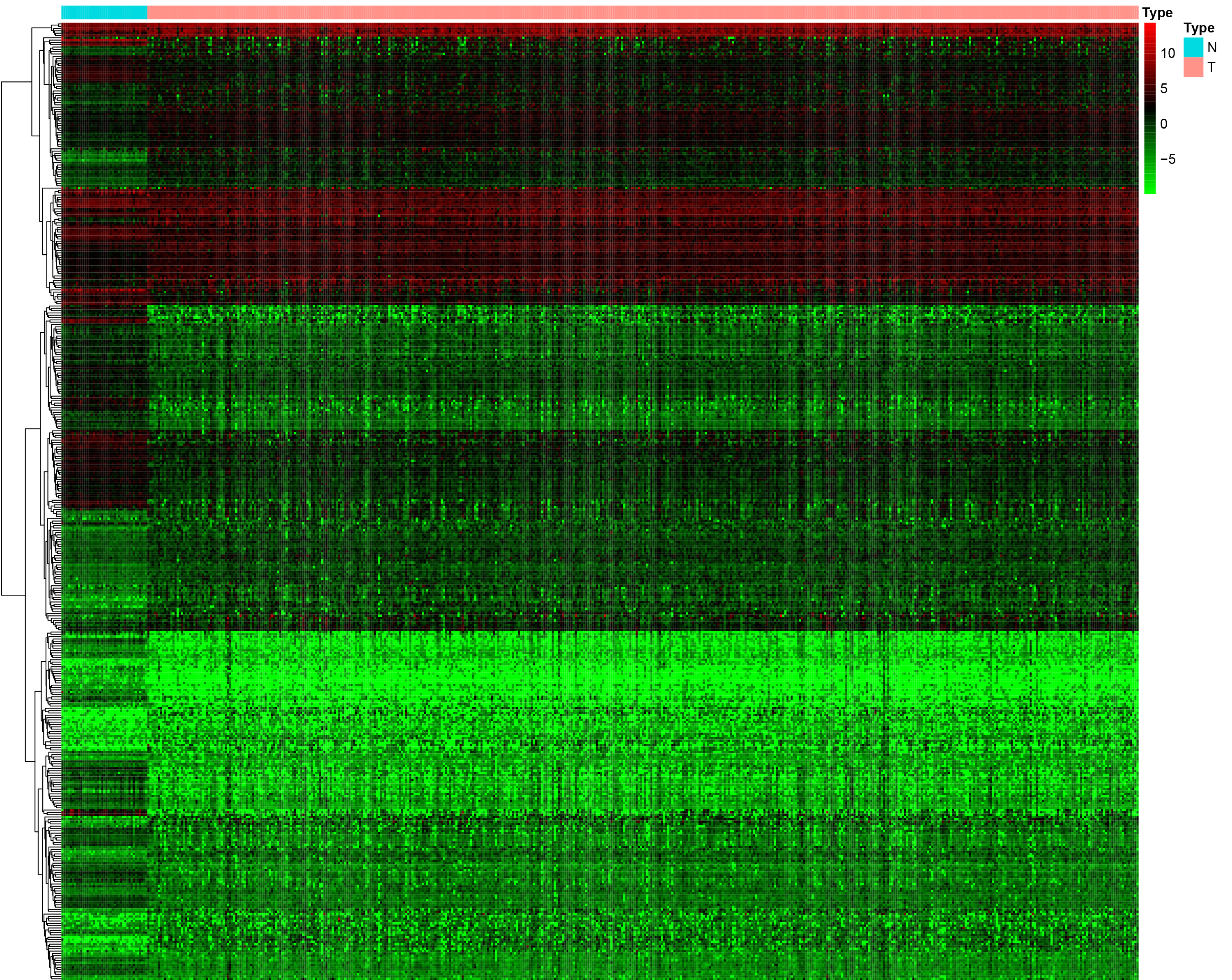

Supplement: Figure S1 [file peerj-09-10812-s001.png]

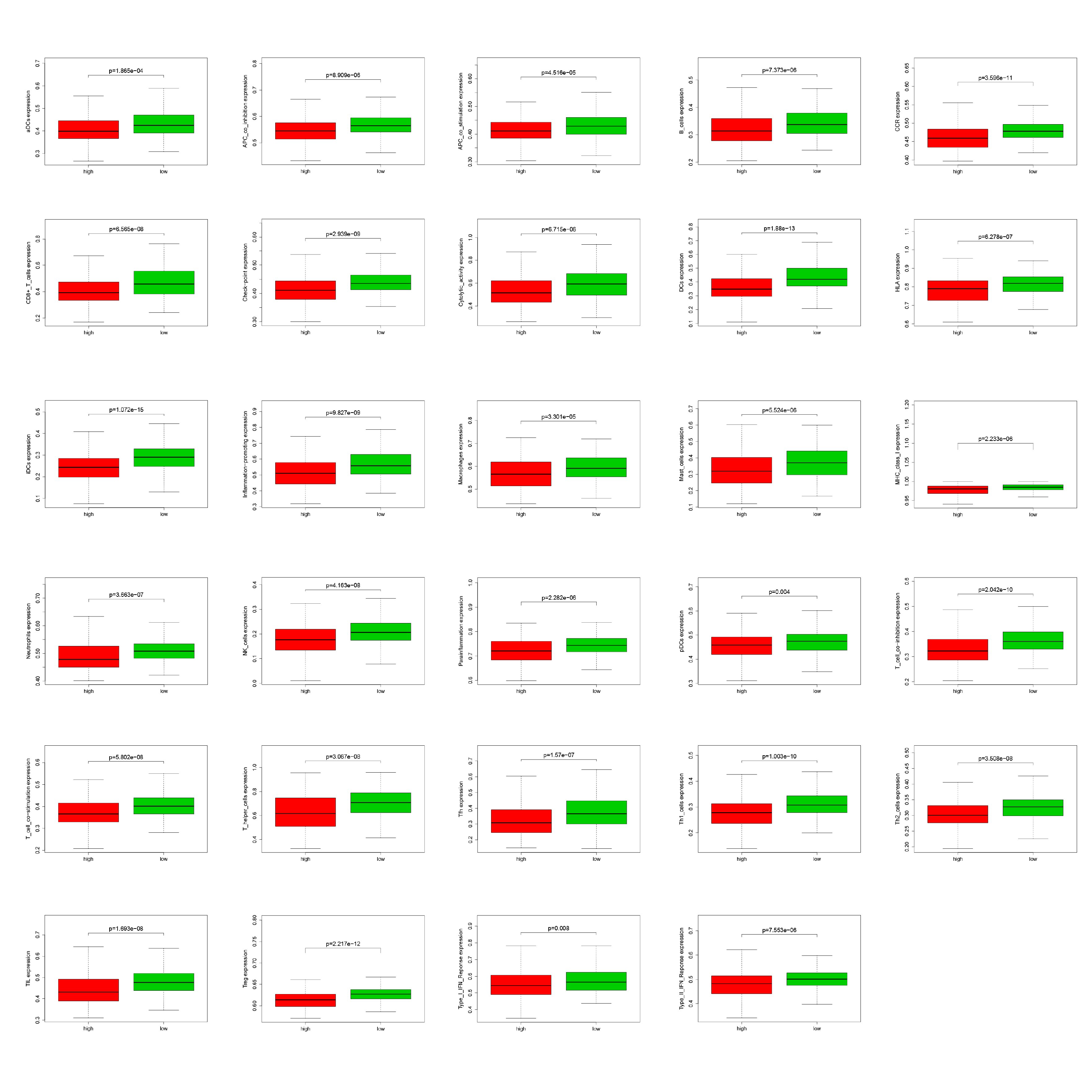

Supplement: Figure S2 — Low-risk group showed significantly higher enrichment level of 29 known immune signatures than high-risk group (Mann-Whitney U test, P < 0.05). [file peerj-09-10812-s002.png]
