# Supplementary material for: Construction of an immune-related signature with prognostic value for colon cancer
Source: PeerJ. 2021 May 5;9:e10812. doi: 10.7717/peerj.10812 (PMC8106397; doi:10.7717/peerj.10812)
Supplement: Table S3 — We exerted Wilcoxon signed-ranked tests to screen immune-related genes (—FC (Fold change) —> 1, P < 0.05 and FDR < 0.25) between normal tissue samples and primary tumor tissue samples from TCGA. FDR, false discovery rate; FC, fold change; TCGA, The Cancer Genome Atlas. [file peerj-09-10812-s005.docx]

| Table S3 Screening of differentially expressed immune-related genes. | | | | | |
| --- | --- | --- | --- | --- | --- |
| ID | **conMean** | **treatMean** | **logFC** | **pValue** | **FDR** |
| MCHR2 | 0.0359611 | 0.0059655 | -2.59171 | 4.64E-33 | 6.67E-30 |
| SLC10A2 | 4.18078 | 0.0275126 | -7.247536 | 8.62E-32 | 1.05E-28 |
| AVPR1B | 0.1189847 | 0.0122094 | -3.284709 | 8.45E-30 | 7.42E-27 |
| CMTM5 | 0.3573002 | 0.0170968 | -4.385342 | 1.01E-27 | 5.01E-25 |
| PMP2 | 0.3632588 | 0.0352251 | -3.366322 | 2.85E-27 | 1.22E-24 |
| PCSK2 | 0.8528919 | 0.0450349 | -4.243248 | 1.14E-26 | 3.83E-24 |
| ELANE | 0.7874192 | 0.07102 | -3.470834 | 1.56E-26 | 5.04E-24 |
| GLP2R | 1.971595 | 0.1478516 | -3.737141 | 2.56E-26 | 7.09E-24 |
| ESM1 | 0.0481186 | 2.985192 | 5.9550849 | 2.66E-26 | 7.21E-24 |
| IL6R | 12.39613 | 2.043049 | -2.601094 | 2.89E-26 | 7.45E-24 |
| RXRG | 0.7206077 | 0.0512368 | -3.813963 | 3.46E-26 | 8.54E-24 |
| BMP3 | 11.05522 | 0.3311532 | -5.061086 | 3.52E-26 | 8.56E-24 |
| INHBA | 0.1291431 | 6.724236 | 5.7023279 | 4.89E-26 | 1.02E-23 |
| PYY | 73.74668 | 1.308599 | -5.816483 | 5.41E-26 | 1.08E-23 |
| SEMA6D | 6.56828 | 0.8744876 | -2.909006 | 9.94E-26 | 1.54E-23 |
| GUCA2A | 994.9734 | 27.9527 | -5.153598 | 1.08E-25 | 1.60E-23 |
| HTR3B | 0.0255553 | 0.0064295 | -1.990844 | 1.42E-25 | 1.93E-23 |
| CHGA | 92.38148 | 4.436465 | -4.380121 | 1.52E-25 | 2.00E-23 |
| GCG | 22.07889 | 2.549102 | -3.114607 | 1.87E-25 | 2.34E-23 |
| GREM2 | 11.43577 | 0.931 | -3.618628 | 2.08E-25 | 2.44E-23 |
| NR3C2 | 19.13975 | 3.375167 | -2.503541 | 2.21E-25 | 2.56E-23 |
| SST | 26.97694 | 0.8846026 | -4.930553 | 2.96E-25 | 3.10E-23 |
| ZC3HAV1L | 1.030706 | 4.983825 | 2.2736212 | 3.74E-25 | 3.70E-23 |
| CHP2 | 147.8248 | 10.00272 | -3.885424 | 5.71E-25 | 4.78E-23 |
| ULBP2 | 0.0822109 | 1.858212 | 4.4984417 | 6.41E-25 | 5.04E-23 |
| EDN3 | 24.2212 | 2.599488 | -3.219971 | 6.55E-25 | 5.08E-23 |
| CMA1 | 2.927801 | 0.2504447 | -3.547254 | 7.53E-25 | 5.56E-23 |
| S100A2 | 0.3447439 | 7.141855 | 4.372702 | 7.51E-25 | 5.56E-23 |
| TRIM27 | 5.961544 | 11.95076 | 1.0033446 | 8.14E-25 | 5.74E-23 |
| SEMA6A | 10.49262 | 2.320264 | -2.177014 | 1.72E-24 | 1.02E-22 |
| MET | 6.455994 | 26.85181 | 2.0563082 | 1.76E-24 | 1.03E-22 |
| APLN | 0.3337789 | 3.295931 | 3.3037211 | 1.74E-24 | 1.03E-22 |
| VIP | 32.02157 | 3.103312 | -3.367163 | 1.80E-24 | 1.05E-22 |
| PTN | 8.123193 | 1.450918 | -2.485081 | 1.86E-24 | 1.07E-22 |
| PPY | 0.6639751 | 0.034765 | -4.25542 | 1.97E-24 | 1.13E-22 |
| LIFR | 2.359752 | 0.2993259 | -2.978846 | 2.23E-24 | 1.24E-22 |
| NR5A2 | 6.82378 | 1.487008 | -2.198158 | 3.13E-24 | 1.59E-22 |
| UCN2 | 0.0155514 | 0.4441699 | 4.8359962 | 4.22E-24 | 2.00E-22 |
| SEMA3E | 0.7402018 | 0.0767034 | -3.270557 | 6.53E-24 | 2.80E-22 |
| CCL23 | 3.357538 | 0.427732 | -2.972624 | 7.18E-24 | 2.98E-22 |
| FGFRL1 | 4.424087 | 23.96059 | 2.4372116 | 8.36E-24 | 3.32E-22 |
| NPY | 1.090266 | 0.1055797 | -3.368275 | 1.01E-23 | 3.77E-22 |
| CLEC4M | 0.0583662 | 0.0121107 | -2.268855 | 1.13E-23 | 4.07E-22 |
| CNTFR | 9.54465 | 0.7389449 | -3.691154 | 1.29E-23 | 4.43E-22 |
| CTSG | 6.076998 | 0.7129372 | -3.091512 | 1.39E-23 | 4.66E-22 |
| CCL14 | 0.9509933 | 0.2271006 | -2.066104 | 1.72E-23 | 5.51E-22 |
| ADCYAP1R1 | 0.6661769 | 0.0522684 | -3.671894 | 1.90E-23 | 5.96E-22 |
| OGN | 12.08054 | 1.111031 | -3.442714 | 2.01E-23 | 6.15E-22 |
| IL23A | 0.3608322 | 3.159239 | 3.1301769 | 2.15E-23 | 6.49E-22 |
| ANGPTL5 | 0.1469809 | 0.0173787 | -3.080233 | 2.29E-23 | 6.81E-22 |
| ANGPTL7 | 1.532729 | 0.1376376 | -3.477156 | 3.07E-23 | 8.44E-22 |
| OXTR | 0.0737882 | 0.5919286 | 3.0039606 | 3.60E-23 | 9.74E-22 |
| CXCL12 | 21.31919 | 4.078559 | -2.386021 | 4.29E-23 | 1.11E-21 |
| TLR3 | 5.552192 | 1.531026 | -1.858559 | 5.00E-23 | 1.25E-21 |
| VEGFA | 4.312489 | 13.31849 | 1.6268382 | 5.40E-23 | 1.33E-21 |
| TNFRSF12A | 5.813192 | 33.71729 | 2.5360859 | 5.83E-23 | 1.41E-21 |
| HSP90AB1 | 232.6772 | 541.2067 | 1.21785 | 6.22E-23 | 1.49E-21 |
| PTGER4 | 17.32887 | 6.463287 | -1.422838 | 7.17E-23 | 1.68E-21 |
| NMB | 1.79889 | 7.480618 | 2.0560503 | 7.74E-23 | 1.81E-21 |
| ULBP1 | 0.0191055 | 0.3306264 | 4.1131395 | 8.43E-23 | 1.95E-21 |
| GDF15 | 10.68297 | 101.8464 | 3.2530092 | 9.11E-23 | 2.07E-21 |
| AEN | 3.044121 | 8.577518 | 1.4945347 | 9.51E-23 | 2.14E-21 |
| S100A11 | 167.9118 | 676.5794 | 2.010556 | 9.83E-23 | 2.20E-21 |
| FABP2 | 25.21012 | 3.116326 | -3.016085 | 1.05E-22 | 2.30E-21 |
| TNFRSF13B | 1.136596 | 0.128902 | -3.140373 | 1.13E-22 | 2.45E-21 |
| HTR3C | 0.4347696 | 0.0967331 | -2.168169 | 1.30E-22 | 2.76E-21 |
| SECTM1 | 64.12477 | 9.248894 | -2.793529 | 1.73E-22 | 3.53E-21 |
| FGFR2 | 9.244901 | 2.322799 | -1.992793 | 2.49E-22 | 4.87E-21 |
| HTR3E | 0.7132654 | 0.0763135 | -3.224428 | 2.51E-22 | 4.90E-21 |
| PRKCB | 2.747982 | 0.646909 | -2.086738 | 2.63E-22 | 5.10E-21 |
| CMTM8 | 5.336572 | 14.20263 | 1.4121733 | 3.22E-22 | 6.09E-21 |
| IL11 | 0.0779939 | 1.911324 | 4.6150683 | 3.67E-22 | 6.78E-21 |
| PLXNA1 | 3.1073 | 8.468234 | 1.4463995 | 4.40E-22 | 7.89E-21 |
| LIF | 2.52545 | 9.802446 | 1.9566013 | 4.79E-22 | 8.34E-21 |
| ULBP3 | 0.3846771 | 1.788019 | 2.2166422 | 5.06E-22 | 8.71E-21 |
| CDK4 | 14.70668 | 37.52964 | 1.3515585 | 5.22E-22 | 8.93E-21 |
| NRG2 | 0.2831951 | 0.0450264 | -2.652954 | 5.47E-22 | 9.28E-21 |
| SSTR2 | 0.6818657 | 0.1543918 | -2.142892 | 6.06E-22 | 1.01E-20 |
| RAET1L | 0.0705623 | 1.203264 | 4.0919126 | 6.56E-22 | 1.08E-20 |
| TNFRSF17 | 11.74497 | 1.38808 | -3.08088 | 7.23E-22 | 1.17E-20 |
| BMP5 | 2.859085 | 0.5261139 | -2.442106 | 7.82E-22 | 1.25E-20 |
| ANGPTL1 | 6.874726 | 0.669376 | -3.360413 | 8.51E-22 | 1.34E-20 |
| CD209 | 6.77046 | 1.475317 | -2.198229 | 1.00E-21 | 1.53E-20 |
| INSL5 | 44.25744 | 0.5927975 | -6.222237 | 1.23E-21 | 1.83E-20 |
| TNFSF15 | 0.842547 | 2.490465 | 1.5635863 | 1.58E-21 | 2.28E-20 |
| TNFRSF10B | 7.268095 | 19.5068 | 1.4243282 | 1.89E-21 | 2.67E-20 |
| STC2 | 0.3501523 | 4.92824 | 3.8150182 | 1.99E-21 | 2.80E-20 |
| SCG2 | 5.54514 | 1.330376 | -2.05939 | 2.35E-21 | 3.24E-20 |
| ADRB2 | 0.7988016 | 0.238227 | -1.7455 | 2.93E-21 | 3.94E-20 |
| THRB | 3.768413 | 0.8188412 | -2.202301 | 5.21E-21 | 6.56E-20 |
| PSMD14 | 5.928099 | 12.40038 | 1.064743 | 5.32E-21 | 6.68E-20 |
| PLAU | 5.703129 | 26.66465 | 2.2251029 | 6.69E-21 | 8.20E-20 |
| BIRC5 | 6.668028 | 20.39776 | 1.6130784 | 6.76E-21 | 8.27E-20 |
| RNASEL | 4.749522 | 2.177903 | -1.124842 | 7.58E-21 | 9.15E-20 |
| LCN6 | 0.3296335 | 0.0590508 | -2.480834 | 7.71E-21 | 9.28E-20 |
| IL16 | 2.177166 | 0.7157953 | -1.604832 | 8.15E-21 | 9.73E-20 |
| CD1D | 3.36812 | 1.137439 | -1.566154 | 8.76E-21 | 1.04E-19 |
| LMBR1 | 2.789798 | 5.769164 | 1.0482015 | 9.13E-21 | 1.08E-19 |
| ACVRL1 | 35.30334 | 12.38856 | -1.510797 | 1.00E-20 | 1.17E-19 |
| PTH1R | 0.7203432 | 0.2006365 | -1.8441 | 1.38E-20 | 1.56E-19 |
| CXCL3 | 2.828289 | 24.19823 | 3.0969 | 1.82E-20 | 1.99E-19 |
| TG | 0.0510698 | 1.040924 | 4.3492495 | 1.92E-20 | 2.09E-19 |
| NOX4 | 0.0465297 | 0.5211617 | 3.4855059 | 2.29E-20 | 2.44E-19 |
| SEMA3G | 4.200224 | 1.191925 | -1.817172 | 2.86E-20 | 2.99E-19 |
| BID | 6.468851 | 14.05085 | 1.1190762 | 3.01E-20 | 3.13E-19 |
| BMP2 | 16.49741 | 4.914268 | -1.747191 | 3.34E-20 | 3.43E-19 |
| CCL28 | 25.20241 | 6.337419 | -1.991595 | 3.73E-20 | 3.79E-19 |
| CCL15 | 8.270795 | 3.288455 | -1.330616 | 4.39E-20 | 4.34E-19 |
| NR3C1 | 4.742841 | 1.787216 | -1.408037 | 5.44E-20 | 5.30E-19 |
| PTK2B | 15.42686 | 7.09564 | -1.120439 | 5.96E-20 | 5.75E-19 |
| PLCG2 | 2.656086 | 0.8288417 | -1.680133 | 6.40E-20 | 6.10E-19 |
| PDGFD | 3.599055 | 1.318358 | -1.448876 | 7.30E-20 | 6.90E-19 |
| TNFSF9 | 0.4777183 | 6.685836 | 3.8068759 | 7.37E-20 | 6.96E-19 |
| BMP6 | 2.593084 | 0.7609869 | -1.768726 | 7.60E-20 | 7.16E-19 |
| ADIPOQ | 1.761614 | 0.6078521 | -1.535106 | 7.80E-20 | 7.35E-19 |
| TFR2 | 0.1469506 | 1.098406 | 2.9020083 | 8.07E-20 | 7.59E-19 |
| SEMA3D | 0.6119203 | 0.1925295 | -1.668264 | 1.06E-19 | 9.69E-19 |
| GDNF | 0.9324708 | 0.2311718 | -2.012093 | 1.23E-19 | 1.11E-18 |
| CXCL1 | 8.799406 | 70.87381 | 3.0097746 | 1.38E-19 | 1.23E-18 |
| F2RL1 | 70.97299 | 32.82457 | -1.112494 | 1.52E-19 | 1.35E-18 |
| AGTR1 | 1.838394 | 0.1716838 | -3.42062 | 2.15E-19 | 1.85E-18 |
| LGR4 | 24.81154 | 11.93884 | -1.055348 | 2.23E-19 | 1.91E-18 |
| NR1H4 | 4.862076 | 0.3812188 | -3.672881 | 2.54E-19 | 2.15E-18 |
| TNFSF10 | 51.5492 | 20.34346 | -1.341385 | 3.35E-19 | 2.77E-18 |
| AZGP1 | 3.252323 | 29.96434 | 3.2037041 | 3.42E-19 | 2.82E-18 |
| CCL21 | 64.63659 | 17.64821 | -1.872829 | 3.52E-19 | 2.90E-18 |
| UCN | 0.2300035 | 1.184531 | 2.3645882 | 3.54E-19 | 2.91E-18 |
| CYSLTR1 | 0.8332887 | 0.2981303 | -1.482874 | 3.56E-19 | 2.92E-18 |
| FABP4 | 11.78238 | 4.45365 | -1.403571 | 3.73E-19 | 3.06E-18 |
| PGR | 0.3282146 | 0.0755827 | -2.118511 | 4.05E-19 | 3.28E-18 |
| FLT3 | 0.3806817 | 0.1380172 | -1.463737 | 4.23E-19 | 3.41E-18 |
| ADRM1 | 39.46523 | 82.97851 | 1.0721555 | 4.51E-19 | 3.62E-18 |
| CST4 | 0.0044228 | 0.7410719 | 7.3884965 | 4.76E-19 | 3.81E-18 |
| HTR3A | 0.5725668 | 0.1192824 | -2.263063 | 5.13E-19 | 4.07E-18 |
| IL20RA | 2.480068 | 6.772554 | 1.4493204 | 6.13E-19 | 4.80E-18 |
| JAG2 | 1.868187 | 7.742687 | 2.0511954 | 6.57E-19 | 5.12E-18 |
| SLC22A17 | 3.631795 | 1.196293 | -1.602112 | 6.76E-19 | 5.25E-18 |
| CD79A | 31.10105 | 6.274652 | -2.309356 | 7.04E-19 | 5.43E-18 |
| EDNRB | 4.626768 | 1.962477 | -1.237329 | 7.04E-19 | 5.43E-18 |
| GHR | 1.873682 | 0.4796036 | -1.965962 | 8.74E-19 | 6.64E-18 |
| OLR1 | 0.150179 | 2.463243 | 4.0358037 | 9.36E-19 | 7.06E-18 |
| STAB2 | 0.3397557 | 0.0925218 | -1.876633 | 9.64E-19 | 7.26E-18 |
| IGF1 | 0.3255615 | 0.1096832 | -1.569588 | 1.09E-18 | 8.15E-18 |
| VIPR1 | 14.8012 | 4.956143 | -1.578425 | 1.12E-18 | 8.30E-18 |
| CMTM7 | 2.104607 | 6.036157 | 1.5200795 | 1.23E-18 | 9.08E-18 |
| S100B | 8.329876 | 1.878353 | -2.148827 | 1.27E-18 | 9.31E-18 |
| HMOX1 | 35.27644 | 12.05835 | -1.548672 | 1.32E-18 | 9.65E-18 |
| COLEC12 | 2.887114 | 0.9811516 | -1.55708 | 2.00E-18 | 1.42E-17 |
| UCN3 | 3.321638 | 1.124223 | -1.562966 | 2.94E-18 | 2.01E-17 |
| GNAI1 | 7.190612 | 2.977549 | -1.271989 | 4.14E-18 | 2.77E-17 |
| HSPA2 | 12.56611 | 4.827454 | -1.380204 | 4.43E-18 | 2.94E-17 |
| LCN10 | 0.0820314 | 0.0160888 | -2.350117 | 4.48E-18 | 2.98E-17 |
| PTX3 | 1.19176 | 0.4132383 | -1.528047 | 4.52E-18 | 2.99E-17 |
| NGFR | 2.392101 | 0.7532368 | -1.667103 | 4.56E-18 | 3.02E-17 |
| KL | 0.7069571 | 0.2878075 | -1.296518 | 4.60E-18 | 3.04E-17 |
| CCR10 | 0.8665538 | 0.2647651 | -1.710576 | 5.17E-18 | 3.39E-17 |
| PTGDR | 3.897455 | 1.24976 | -1.640881 | 5.53E-18 | 3.61E-17 |
| CCL13 | 15.47024 | 2.896812 | -2.416958 | 5.57E-18 | 3.63E-17 |
| A2M | 100.1035 | 43.59711 | -1.199188 | 6.89E-18 | 4.40E-17 |
| CD79B | 6.010768 | 1.515137 | -1.988101 | 7.09E-18 | 4.52E-17 |
| S100P | 36.87117 | 207.9413 | 2.4956111 | 7.88E-18 | 4.98E-17 |
| PNOC | 0.9933948 | 0.26394 | -1.912157 | 1.19E-17 | 7.38E-17 |
| CTSS | 119.4432 | 59.30655 | -1.010062 | 1.61E-17 | 9.75E-17 |
| PAK3 | 0.1337833 | 0.0485014 | -1.463799 | 1.65E-17 | 1.00E-16 |
| STC1 | 0.8833224 | 4.101575 | 2.2151661 | 2.22E-17 | 1.31E-16 |
| NPR1 | 2.613486 | 0.8585569 | -1.60599 | 2.34E-17 | 1.39E-16 |
| PIK3CG | 1.700367 | 0.6085472 | -1.482405 | 3.05E-17 | 1.77E-16 |
| PAEP | 0.0076434 | 5.862431 | 9.5830654 | 3.33E-17 | 1.92E-16 |
| SLC11A1 | 0.2197173 | 1.323452 | 2.5905863 | 3.58E-17 | 2.05E-16 |
| TLR7 | 1.246599 | 0.4230618 | -1.559058 | 3.68E-17 | 2.11E-16 |
| SCTR | 0.2377862 | 0.06735 | -1.819915 | 5.16E-17 | 2.88E-16 |
| CRABP1 | 1.740549 | 0.6018441 | -1.53208 | 5.30E-17 | 2.95E-16 |
| OSTN | 0.0138638 | 0.0065289 | -1.086406 | 5.71E-17 | 3.16E-16 |
| PGC | 0.0338906 | 1.264261 | 5.221267 | 6.48E-17 | 3.56E-16 |
| DES | 805.2658 | 71.04126 | -3.502736 | 7.00E-17 | 3.82E-16 |
| FGF19 | 0.1055386 | 1.499664 | 3.8287964 | 7.08E-17 | 3.85E-16 |
| SSTR1 | 4.023413 | 1.723148 | -1.223373 | 7.33E-17 | 3.99E-16 |
| CCL8 | 7.302036 | 1.504673 | -2.278849 | 7.40E-17 | 4.02E-16 |
| IL1R2 | 13.70693 | 4.085494 | -1.746323 | 1.15E-16 | 6.12E-16 |
| LTBP4 | 37.61453 | 16.12366 | -1.222111 | 1.22E-16 | 6.44E-16 |
| PLXNA3 | 2.355247 | 5.667222 | 1.2667633 | 1.46E-16 | 7.64E-16 |
| BMP4 | 6.767025 | 26.79302 | 1.9852633 | 1.81E-16 | 9.32E-16 |
| TEK | 2.918494 | 1.314975 | -1.150189 | 1.93E-16 | 9.89E-16 |
| CD48 | 8.060042 | 2.719216 | -1.567596 | 1.98E-16 | 1.01E-15 |
| KIR2DL4 | 0.8780446 | 0.2483642 | -1.821837 | 2.01E-16 | 1.03E-15 |
| AMELX | 0.0085814 | 0.4556193 | 5.7304775 | 2.11E-16 | 1.08E-15 |
| CXCR5 | 0.1665648 | 0.0302633 | -2.460444 | 2.41E-16 | 1.22E-15 |
| PLXNA2 | 9.050797 | 4.342028 | -1.059676 | 2.63E-16 | 1.32E-15 |
| CXCL2 | 3.438389 | 19.33565 | 2.4914587 | 2.85E-16 | 1.43E-15 |
| SH2D1B | 0.434217 | 0.1550627 | -1.485565 | 2.90E-16 | 1.45E-15 |
| CMKLR1 | 3.736704 | 1.562346 | -1.258052 | 3.01E-16 | 1.50E-15 |
| CSRP1 | 93.79849 | 23.12054 | -2.020389 | 3.27E-16 | 1.62E-15 |
| AHNAK | 67.23007 | 27.12603 | -1.309429 | 3.99E-16 | 1.96E-15 |
| NCR2 | 0.1303783 | 0.0257852 | -2.338092 | 4.09E-16 | 2.01E-15 |
| CXCL5 | 0.4109108 | 18.24542 | 5.472565 | 4.33E-16 | 2.12E-15 |
| IL5RA | 0.1082266 | 0.0361377 | -1.582478 | 4.55E-16 | 2.22E-15 |
| CCL19 | 20.32606 | 4.919756 | -2.046672 | 5.23E-16 | 2.53E-15 |
| IFITM1 | 83.89829 | 284.1856 | 1.76012 | 5.38E-16 | 2.60E-15 |
| APOBEC3A | 2.087771 | 0.3660699 | -2.511772 | 6.67E-16 | 3.18E-15 |
| NRG3 | 0.049242 | 0.0159667 | -1.62482 | 6.71E-16 | 3.20E-15 |
| MIF | 22.03 | 56.21578 | 1.3515058 | 7.71E-16 | 3.66E-15 |
| AGTR2 | 0.0310936 | 0.0079302 | -1.971186 | 8.10E-16 | 3.83E-15 |
| IL2 | 0.0710532 | 0.022683 | -1.647287 | 1.06E-15 | 4.92E-15 |
| SFTPD | 0.2642395 | 0.1209898 | -1.12696 | 1.40E-15 | 6.42E-15 |
| NRG4 | 0.2513387 | 0.1026937 | -1.291285 | 1.49E-15 | 6.80E-15 |
| MASP1 | 2.203328 | 0.2805106 | -2.973557 | 1.59E-15 | 7.21E-15 |
| DEFB1 | 24.74396 | 6.517938 | -1.924589 | 2.14E-15 | 9.61E-15 |
| IL1A | 0.1644398 | 1.575974 | 3.2606126 | 2.22E-15 | 9.93E-15 |
| LGR5 | 2.128169 | 17.0095 | 2.9986566 | 2.24E-15 | 1.00E-14 |
| PLCG1 | 4.582236 | 10.13129 | 1.1446939 | 2.43E-15 | 1.08E-14 |
| EDNRA | 0.8874522 | 3.758049 | 2.0822424 | 2.82E-15 | 1.24E-14 |
| BTK | 2.274743 | 0.9241513 | -1.299503 | 2.97E-15 | 1.31E-14 |
| CSF2 | 0.0847387 | 0.9625168 | 3.5057183 | 3.69E-15 | 1.60E-14 |
| NRG1 | 0.5610452 | 0.227305 | -1.303487 | 4.11E-15 | 1.77E-14 |
| CD22 | 2.501807 | 0.5360301 | -2.222585 | 4.40E-15 | 1.89E-14 |
| LTB4R | 0.6416058 | 1.927909 | 1.5872776 | 4.89E-15 | 2.08E-14 |
| TNFRSF10C | 0.3560494 | 1.177251 | 1.725273 | 5.82E-15 | 2.46E-14 |
| SPP1 | 4.504047 | 82.43657 | 4.1939908 | 6.75E-15 | 2.83E-14 |
| PDF | 1.638202 | 4.374643 | 1.4170516 | 6.81E-15 | 2.86E-14 |
| AGT | 3.07647 | 13.23187 | 2.1046694 | 7.42E-15 | 3.10E-14 |
| IL1RAP | 0.4009418 | 0.9346939 | 1.2211012 | 8.45E-15 | 3.50E-14 |
| FAS | 9.642524 | 4.791014 | -1.00908 | 8.98E-15 | 3.71E-14 |
| SLIT2 | 1.818188 | 0.6337393 | -1.52054 | 9.21E-15 | 3.80E-14 |
| CXCL11 | 1.614603 | 10.5608 | 2.709467 | 9.96E-15 | 4.09E-14 |
| IL10RA | 5.550291 | 2.509364 | -1.145242 | 1.06E-14 | 4.33E-14 |
| MC1R | 0.2526759 | 0.7282278 | 1.5271019 | 1.27E-14 | 5.13E-14 |
| CXCL16 | 18.91022 | 39.56747 | 1.0651484 | 1.29E-14 | 5.22E-14 |
| XCR1 | 0.417457 | 0.2036284 | -1.035689 | 1.58E-14 | 6.35E-14 |
| FGF10 | 0.8228869 | 0.235541 | -1.804716 | 1.58E-14 | 6.37E-14 |
| CR2 | 5.260972 | 0.9843955 | -2.418019 | 1.62E-14 | 6.51E-14 |
| TNFRSF10A | 3.930626 | 8.181619 | 1.0576272 | 1.70E-14 | 6.77E-14 |
| MAPT | 0.498098 | 0.1313216 | -1.923325 | 1.78E-14 | 7.11E-14 |
| EBI3 | 3.007329 | 1.272559 | -1.24075 | 1.85E-14 | 7.35E-14 |
| AMH | 0.0883084 | 1.694327 | 4.262017 | 2.30E-14 | 9.04E-14 |
| FGF9 | 0.4968105 | 0.2183286 | -1.186195 | 2.44E-14 | 9.55E-14 |
| RFXAP | 1.722551 | 3.493837 | 1.0202652 | 3.40E-14 | 1.30E-13 |
| CX3CR1 | 0.7651948 | 0.2972764 | -1.364022 | 3.49E-14 | 1.34E-13 |
| TDGF1 | 0.6889093 | 6.073362 | 3.1401093 | 3.55E-14 | 1.36E-13 |
| CD1C | 2.407549 | 0.8551646 | -1.493291 | 5.10E-14 | 1.92E-13 |
| TACR1 | 0.3537731 | 0.1138388 | -1.635832 | 5.48E-14 | 2.06E-13 |
| ILK | 7.645702 | 3.491947 | -1.130617 | 5.55E-14 | 2.08E-13 |
| CSF1R | 14.96116 | 6.759174 | -1.146303 | 6.72E-14 | 2.50E-13 |
| LEPR | 1.232324 | 0.5724552 | -1.106147 | 6.89E-14 | 2.56E-13 |
| FGF18 | 0.2958703 | 1.145843 | 1.9533727 | 6.95E-14 | 2.57E-13 |
| ACVR1C | 1.584872 | 0.7449388 | -1.089172 | 7.62E-14 | 2.81E-13 |
| PGF | 0.6841821 | 1.916833 | 1.4862725 | 8.14E-14 | 2.99E-13 |
| FAM3D | 291.0347 | 136.196 | -1.095507 | 1.31E-13 | 4.69E-13 |
| XCL2 | 1.198648 | 0.4171737 | -1.522688 | 1.39E-13 | 4.97E-13 |
| WNT5A | 1.233824 | 3.854204 | 1.6432963 | 1.42E-13 | 5.07E-13 |
| CYSLTR2 | 0.3200444 | 0.1570588 | -1.026968 | 1.53E-13 | 5.43E-13 |
| LCN12 | 0.3646639 | 1.668078 | 2.1935479 | 1.61E-13 | 5.70E-13 |
| TNFSF12 | 12.87857 | 5.993303 | -1.103549 | 2.19E-13 | 7.66E-13 |
| EDN2 | 4.376849 | 0.6609414 | -2.727298 | 2.50E-13 | 8.66E-13 |
| F2R | 4.752981 | 10.6257 | 1.160653 | 3.43E-13 | 1.17E-12 |
| RSAD2 | 6.233205 | 2.383779 | -1.386723 | 3.67E-13 | 1.25E-12 |
| CD40LG | 1.058795 | 0.5039748 | -1.071 | 3.82E-13 | 1.30E-12 |
| PPBP | 0.1720481 | 95.76959 | 9.1206119 | 3.84E-13 | 1.30E-12 |
| CSF2RB | 5.825672 | 2.868699 | -1.022028 | 4.90E-13 | 1.64E-12 |
| S100A3 | 0.4934133 | 1.989545 | 2.0115698 | 4.90E-13 | 1.64E-12 |
| INHBE | 0.0897154 | 0.2487751 | 1.4714147 | 6.24E-13 | 2.07E-12 |
| FGF16 | 0.0329669 | 0.007376 | -2.160099 | 9.98E-13 | 3.25E-12 |
| GH1 | 0.0363979 | 0.0151231 | -1.267099 | 1.16E-12 | 3.74E-12 |
| TUBB3 | 0.2182064 | 0.773685 | 1.8260527 | 1.25E-12 | 4.03E-12 |
| CCR9 | 0.2046344 | 0.0743811 | -1.460041 | 1.30E-12 | 4.19E-12 |
| AR | 0.426494 | 0.1651362 | -1.368869 | 1.31E-12 | 4.22E-12 |
| NOS1 | 0.3753676 | 0.0495785 | -2.920517 | 1.52E-12 | 4.87E-12 |
| CD19 | 2.083104 | 0.6024303 | -1.789869 | 1.61E-12 | 5.14E-12 |
| CXCL17 | 0.0347126 | 1.507901 | 5.4409404 | 1.89E-12 | 5.99E-12 |
| FGFR4 | 10.22241 | 25.92968 | 1.3428683 | 1.90E-12 | 6.02E-12 |
| INPP5D | 3.653106 | 8.197532 | 1.1660662 | 2.52E-12 | 7.87E-12 |
| PTGDS | 17.47546 | 7.31409 | -1.25658 | 3.04E-12 | 9.41E-12 |
| CCL11 | 10.93649 | 4.572417 | -1.258121 | 5.23E-12 | 1.58E-11 |
| FGF2 | 1.483024 | 0.6241661 | -1.24854 | 6.25E-12 | 1.87E-11 |
| NOD2 | 0.4528952 | 1.33387 | 1.5583693 | 7.01E-12 | 2.09E-11 |
| GPR17 | 0.5411986 | 0.2223497 | -1.283328 | 7.38E-12 | 2.19E-11 |
| FAM19A4 | 0.0926403 | 0.0230838 | -2.004757 | 7.51E-12 | 2.23E-11 |
| OSM | 0.6606696 | 3.540034 | 2.4217626 | 8.30E-12 | 2.45E-11 |
| CCR8 | 0.0747407 | 0.3801886 | 2.34675 | 8.41E-12 | 2.48E-11 |
| NDRG1 | 88.20901 | 39.41104 | -1.162326 | 9.02E-12 | 2.65E-11 |
| ACVR2B | 0.4811118 | 0.9874939 | 1.0373997 | 9.81E-12 | 2.88E-11 |
| MTNR1A | 0.6828563 | 0.3166434 | -1.108723 | 1.04E-11 | 3.02E-11 |
| FGA | 0.0415762 | 0.2201887 | 2.4049103 | 1.16E-11 | 3.35E-11 |
| CAMP | 0.2770641 | 0.1167672 | -1.246584 | 1.18E-11 | 3.42E-11 |
| GZMB | 2.26311 | 10.81795 | 2.2570487 | 1.56E-11 | 4.47E-11 |
| SEMA3F | 4.139428 | 9.354338 | 1.1762042 | 2.50E-11 | 7.05E-11 |
| AREG | 15.20101 | 62.86927 | 2.0481882 | 2.74E-11 | 7.68E-11 |
| IL1RN | 1.590795 | 7.472156 | 2.2317765 | 4.30E-11 | 1.19E-10 |
| BACH2 | 0.414281 | 0.1947672 | -1.088859 | 5.82E-11 | 1.58E-10 |
| CCL5 | 31.66894 | 14.07844 | -1.169581 | 6.50E-11 | 1.76E-10 |
| ANGPT1 | 0.7939656 | 0.3821826 | -1.054814 | 6.84E-11 | 1.84E-10 |
| PROCR | 14.82607 | 33.09696 | 1.1585626 | 7.63E-11 | 2.05E-10 |
| NCR3 | 0.6968659 | 0.3082936 | -1.176576 | 8.67E-11 | 2.31E-10 |
| VIPR2 | 0.4387645 | 0.1298916 | -1.756139 | 9.48E-11 | 2.52E-10 |
| OBP2B | 0.0031809 | 0.6726049 | 7.7241979 | 9.48E-11 | 2.52E-10 |
| TSHB | 0.077752 | 0.0283701 | -1.454508 | 1.03E-10 | 2.73E-10 |
| MUC4 | 10.4257 | 4.727874 | -1.140881 | 1.09E-10 | 2.88E-10 |
| KNG1 | 0.5789476 | 0.2034516 | -1.508747 | 1.16E-10 | 3.05E-10 |
| BMP7 | 0.7796325 | 7.913799 | 3.3435043 | 1.21E-10 | 3.18E-10 |
| MSTN | 0.1952671 | 0.0742994 | -1.394026 | 1.22E-10 | 3.20E-10 |
| RAC3 | 1.448224 | 3.867291 | 1.417039 | 1.35E-10 | 3.53E-10 |
| MUC5AC | 0.1970364 | 3.692445 | 4.2280421 | 1.45E-10 | 3.79E-10 |
| CCL26 | 0.2820352 | 1.563767 | 2.4710787 | 1.71E-10 | 4.44E-10 |
| CCL16 | 0.0737713 | 0.0351538 | -1.06938 | 1.87E-10 | 4.82E-10 |
| NPPC | 0.2802043 | 0.1292513 | -1.1163 | 1.89E-10 | 4.88E-10 |
| FAM19A5 | 0.5487886 | 1.545495 | 1.4937461 | 1.99E-10 | 5.12E-10 |
| TNFRSF11B | 1.62657 | 7.097056 | 2.1253877 | 2.50E-10 | 6.36E-10 |
| KIR3DL2 | 0.0724524 | 0.0332485 | -1.123745 | 2.69E-10 | 6.81E-10 |
| VGF | 0.2946189 | 2.561019 | 3.119796 | 3.56E-10 | 8.91E-10 |
| PRKCG | 0.0586256 | 0.7757827 | 3.7260511 | 3.87E-10 | 9.65E-10 |
| CXCL6 | 0.2748898 | 1.645348 | 2.5814677 | 4.01E-10 | 9.98E-10 |
| PENK | 1.112097 | 0.1505278 | -2.885181 | 6.13E-10 | 1.50E-09 |
| IL17C | 0.0341964 | 0.3943121 | 3.5274204 | 7.56E-10 | 1.83E-09 |
| TNFSF11 | 0.4318728 | 1.47911 | 1.7760512 | 8.00E-10 | 1.93E-09 |
| ORM2 | 0.0547472 | 0.5029276 | 3.1994944 | 1.03E-09 | 2.47E-09 |
| PI15 | 1.306048 | 0.6053342 | -1.109404 | 1.51E-09 | 3.57E-09 |
| SERPIND1 | 0.0445922 | 0.6777427 | 3.925873 | 1.93E-09 | 4.51E-09 |
| INHBB | 0.6272822 | 3.12406 | 2.3162356 | 1.94E-09 | 4.53E-09 |
| S100A5 | 0.1821063 | 0.6427704 | 1.8195229 | 2.70E-09 | 6.20E-09 |
| AQP9 | 0.3821747 | 2.428334 | 2.6676629 | 2.76E-09 | 6.33E-09 |
| SPINK5 | 3.152882 | 1.132737 | -1.476859 | 2.89E-09 | 6.61E-09 |
| NR0B2 | 1.792238 | 3.78393 | 1.0781234 | 3.21E-09 | 7.33E-09 |
| SSTR5 | 0.0729847 | 1.415993 | 4.2780778 | 4.01E-09 | 9.06E-09 |
| FGF8 | 0.0094336 | 0.0895333 | 3.2465426 | 5.56E-09 | 1.24E-08 |
| GRP | 0.3513244 | 1.842529 | 2.3908118 | 6.12E-09 | 1.35E-08 |
| MMP12 | 10.63617 | 42.33696 | 1.9929389 | 7.42E-09 | 1.63E-08 |
| EREG | 0.9702212 | 11.56108 | 3.5748185 | 7.52E-09 | 1.65E-08 |
| TNFRSF13C | 1.653638 | 0.5919696 | -1.482049 | 9.82E-09 | 2.13E-08 |
| SAA2 | 0.2890099 | 1.919831 | 2.7317884 | 1.17E-08 | 2.52E-08 |
| CCR7 | 2.593793 | 1.267548 | -1.033023 | 2.30E-08 | 4.82E-08 |
| LCN2 | 91.36289 | 452.4166 | 2.3079716 | 3.37E-08 | 6.97E-08 |
| CCL24 | 16.77799 | 64.32187 | 1.9387394 | 3.43E-08 | 7.10E-08 |
| OASL | 11.7039 | 4.62492 | -1.339489 | 3.61E-08 | 7.44E-08 |
| ROBO2 | 0.0849774 | 0.4001938 | 2.2355481 | 3.84E-08 | 7.90E-08 |
| CSF3 | 0.4093981 | 1.522646 | 1.8950041 | 3.98E-08 | 8.18E-08 |
| HSPA6 | 0.7612418 | 2.500832 | 1.7159814 | 4.71E-08 | 9.61E-08 |
| CTLA4 | 0.4526384 | 1.178004 | 1.3799129 | 5.87E-08 | 1.19E-07 |
| BDNF | 0.048893 | 0.1319802 | 1.4326205 | 7.28E-08 | 1.46E-07 |
| RAET1G | 0.1282834 | 0.3231756 | 1.3329837 | 7.45E-08 | 1.50E-07 |
| IL17B | 0.4035865 | 0.1899308 | -1.087404 | 7.82E-08 | 1.57E-07 |
| HAMP | 0.0495635 | 0.1680542 | 1.7615766 | 8.10E-08 | 1.62E-07 |
| LEP | 0.3964044 | 0.188803 | -1.070091 | 1.07E-07 | 2.11E-07 |
| IFNE | 0.0040141 | 0.101289 | 4.65726 | 2.34E-07 | 4.48E-07 |
| PMCH | 0.0136232 | 0.0710962 | 2.383705 | 2.57E-07 | 4.90E-07 |
| GAST | 0.0095185 | 0.2078709 | 4.4488053 | 2.69E-07 | 5.12E-07 |
| CCL20 | 14.45457 | 48.6082 | 1.7496738 | 2.75E-07 | 5.24E-07 |
| SYTL1 | 1.67788 | 4.221772 | 1.3312091 | 2.88E-07 | 5.48E-07 |
| CALCA | 0.0340357 | 1.742992 | 5.6783721 | 3.52E-07 | 6.64E-07 |
| REG1A | 84.36791 | 350.8846 | 2.0562305 | 3.77E-07 | 7.11E-07 |
| LEAP2 | 1.577076 | 0.6757619 | -1.222665 | 4.17E-07 | 7.83E-07 |
| TNFSF4 | 0.4688346 | 1.406794 | 1.58526 | 4.34E-07 | 8.13E-07 |
| CXCL13 | 13.65864 | 5.207786 | -1.391071 | 4.71E-07 | 8.79E-07 |
| IL1B | 2.99772 | 10.41135 | 1.7962201 | 4.84E-07 | 9.03E-07 |
| LHB | 0.0421995 | 0.2008528 | 2.2508423 | 4.86E-07 | 9.05E-07 |
| NFAT5 | 1.645558 | 3.641292 | 1.1458739 | 6.08E-07 | 1.12E-06 |
| FGF20 | 0.0078432 | 0.5395381 | 6.104136 | 6.08E-07 | 1.12E-06 |
| LTB4R2 | 0.253668 | 0.5525732 | 1.123224 | 7.98E-07 | 1.46E-06 |
| LBP | 0.1910333 | 0.5470385 | 1.5178182 | 8.80E-07 | 1.61E-06 |
| LGR6 | 1.46367 | 6.387877 | 2.1257465 | 1.32E-06 | 2.38E-06 |
| PGLYRP3 | 0.0037363 | 0.1500692 | 5.3278866 | 1.32E-06 | 2.38E-06 |
| OBP2A | 0.0154618 | 0.1446443 | 3.2257336 | 1.35E-06 | 2.41E-06 |
| ORM1 | 0.0479651 | 4.868381 | 6.6653146 | 2.98E-06 | 5.18E-06 |
| IL17A | 0.0387624 | 0.2232496 | 2.5259277 | 3.81E-06 | 6.56E-06 |
| TNFRSF9 | 0.2637606 | 0.5696884 | 1.1109437 | 4.61E-06 | 7.87E-06 |
| FGF17 | 0.0189699 | 0.0712162 | 1.908493 | 5.81E-06 | 9.84E-06 |
| NODAL | 0.0604564 | 0.2890987 | 2.2575951 | 5.99E-06 | 1.01E-05 |
| TPM2 | 176.5175 | 34.41341 | -2.358768 | 7.06E-06 | 1.19E-05 |
| R3HDML | 0.3781628 | 1.485928 | 1.974285 | 9.16E-06 | 1.52E-05 |
| CCL3 | 1.708416 | 4.107054 | 1.265445 | 1.17E-05 | 1.94E-05 |
| PGLYRP4 | 0.0172065 | 0.0975563 | 2.503281 | 1.70E-05 | 2.76E-05 |
| S100A7 | 0.0166227 | 0.8734337 | 5.7154702 | 1.91E-05 | 3.10E-05 |
| PAK6 | 0.0219464 | 0.0441976 | 1.0099856 | 2.36E-05 | 3.78E-05 |
| S100A9 | 33.1544 | 67.48119 | 1.025285 | 2.36E-05 | 3.78E-05 |
| MMP9 | 8.980919 | 25.26218 | 1.4920442 | 2.59E-05 | 4.13E-05 |
| RLN2 | 0.1400929 | 0.4300818 | 1.6182269 | 2.60E-05 | 4.15E-05 |
| LCN1 | 0.0114154 | 0.0868587 | 2.9276851 | 4.22E-05 | 6.60E-05 |
| CCL3L1 | 0.7625542 | 2.059981 | 1.4337195 | 5.09E-05 | 7.91E-05 |
| EPGN | 0.0059123 | 0.0634378 | 3.4235464 | 5.52E-05 | 8.55E-05 |
| IL13RA2 | 0.2537532 | 0.6567067 | 1.3718229 | 6.11E-05 | 9.43E-05 |
| COLEC10 | 0.0509003 | 0.3947625 | 2.9552396 | 6.38E-05 | 9.84E-05 |
| PF4 | 1.117487 | 4.530649 | 2.0194602 | 8.01E-05 | 0.000122413 |
| CD70 | 0.4054317 | 1.542499 | 1.9277382 | 0.0001328 | 0.0001993 |
| INHA | 0.0202825 | 0.1919139 | 3.2421529 | 0.0001552 | 0.000231547 |
| IL1RL2 | 0.4803416 | 1.093534 | 1.1868648 | 0.0002416 | 0.000354552 |
| FCGR3A | 5.736611 | 12.85668 | 1.1642478 | 0.0002533 | 0.00037107 |
| CRABP2 | 2.532384 | 7.9681 | 1.6537393 | 0.0002621 | 0.000383437 |
| CD1A | 0.154375 | 0.3521867 | 1.1899018 | 0.0003015 | 0.000439128 |
| PDIA2 | 0.2704835 | 1.079889 | 1.9972712 | 0.0003159 | 0.00045907 |
| CGB7 | 0.0034555 | 0.0221124 | 2.6778841 | 0.0003682 | 0.000532134 |
| NDP | 0.105513 | 0.4247035 | 2.009035 | 0.000536 | 0.000763113 |
| RETN | 0.1182758 | 0.4880784 | 2.0449575 | 0.0007381 | 0.001037676 |
| NR5A1 | 0.0040395 | 0.0454511 | 3.4920669 | 0.0008112 | 0.001134626 |
| RAET1E | 0.1123124 | 0.2650738 | 1.2388765 | 0.0008855 | 0.001234724 |
| IL13 | 0.0115952 | 0.0391631 | 1.7559671 | 0.0009524 | 0.001323295 |
| OXT | 0.0569119 | 0.1682141 | 1.5634958 | 0.0015205 | 0.00206899 |
| SAA1 | 5.436046 | 21.96908 | 2.0148448 | 0.0015709 | 0.002134657 |
| IFNG | 0.1776145 | 0.3778655 | 1.0891235 | 0.0016281 | 0.002209955 |
| CCL4 | 2.002339 | 4.161516 | 1.0554228 | 0.0026315 | 0.0035003 |
| IL17F | 0.0254731 | 0.1252379 | 2.2976265 | 0.0026545 | 0.003529197 |
| DEFA6 | 38.90482 | 80.76504 | 1.0537821 | 0.002662 | 0.003538205 |
| MPO | 0.0499874 | 0.1110682 | 1.1518099 | 0.00309 | 0.004077911 |
| UTS2 | 0.2263375 | 0.5034628 | 1.1534095 | 0.0031708 | 0.004178608 |
| MARCO | 6.14019 | 2.970554 | -1.047552 | 0.0032607 | 0.004293154 |
| CD1B | 0.1091436 | 0.2519425 | 1.2068666 | 0.0049966 | 0.006458091 |
| DKK1 | 0.0932299 | 1.248865 | 3.743682 | 0.0067427 | 0.008602471 |
| ANGPTL3 | 0.0121998 | 0.1558177 | 3.6749302 | 0.0096556 | 0.01213456 |
| AGRP | 0.0381336 | 0.1064916 | 1.4816038 | 0.0120342 | 0.01499385 |
| PRL | 0.0174512 | 0.0662843 | 1.9253433 | 0.0130272 | 0.01617493 |
| GDF5 | 0.1419599 | 0.0627789 | -1.177132 | 0.015632 | 0.01925929 |
| EPO | 0.0147161 | 0.0393893 | 1.4204052 | 0.0216487 | 0.02629495 |
| RBP4 | 4.301629 | 11.48805 | 1.4171789 | 0.0265153 | 0.03187044 |
| NTF4 | 0.0047625 | 0.0311304 | 2.7085406 | 0.027729 | 0.03325067 |
| OPRD1 | 0.0379723 | 0.168431 | 2.1491371 | 0.028909 | 0.03460857 |
| IL33 | 6.762865 | 14.84423 | 1.1341956 | 0.0294044 | 0.03516372 |
| PTGS2 | 1.486429 | 4.198019 | 1.4978576 | 0.0354234 | 0.04195837 |
