# Supplementary material for: Construction of an immune-related signature with prognostic value for colon cancer
Source: PeerJ. 2021 May 5;9:e10812. doi: 10.7717/peerj.10812 (PMC8106397; doi:10.7717/peerj.10812)
Supplement: Table S4 — We used univariate CoxPH to screen out genes with prognosis capacity (P < 0.05). There were 45 genes selected. CoxPH, Cox proportional hazard model. [file peerj-09-10812-s006.docx]

| Table S4 Further screening of immune-related genes. | | | | |
| --- | --- | --- | --- | --- |
| Id | **HR** | **HR.95L** | **HR.95H** | **pValue** |
| OXTR | 1.39129371 | 1.1536778 | 1.67784988 | 0.000548144 |
| IGF1 | 2.51104071 | 1.4268576 | 4.419029073 | 0.001409951 |
| PTH1R | 1.5648237 | 1.1726969 | 2.088069969 | 0.002347343 |
| PLXNA3 | 1.13999031 | 1.0465517 | 1.241771387 | 0.002675374 |
| EPO | 20.7908586 | 2.8446666 | 151.9544669 | 0.002788741 |
| UCN | 1.41127478 | 1.1208832 | 1.776899238 | 0.003380556 |
| STAB2 | 31.4266742 | 3.1215019 | 316.397642 | 0.003432808 |
| JAG2 | 1.03813296 | 1.012047 | 1.064891275 | 0.003948689 |
| DKK1 | 1.03335586 | 1.0104433 | 1.056787952 | 0.004129577 |
| NGFR | 1.15382029 | 1.0439342 | 1.275273201 | 0.005078875 |
| CD1B | 0.0223364 | 0.0015473 | 0.322435526 | 0.005255858 |
| IL1RL2 | 1.29507483 | 1.0793781 | 1.553875187 | 0.005407025 |
| INHBA | 1.04321442 | 1.0121378 | 1.075245218 | 0.006109046 |
| SEMA3E | 2.84558578 | 1.3353126 | 6.064017108 | 0.006747814 |
| MC1R | 1.51135782 | 1.117483 | 2.044060172 | 0.007339645 |
| NPR1 | 1.38052893 | 1.0902949 | 1.748022555 | 0.00740954 |
| FABP4 | 1.0066082 | 1.0017205 | 1.011519805 | 0.007998528 |
| SFTPD | 24.4268457 | 2.2073567 | 270.3100872 | 0.009172963 |
| F2RL1 | 0.96333523 | 0.9356181 | 0.991873449 | 0.012149412 |
| BDNF | 2.47106875 | 1.1913093 | 5.125604892 | 0.015089675 |
| COLEC12 | 1.27903858 | 1.0486957 | 1.559975621 | 0.015128287 |
| FGF2 | 1.37409394 | 1.0600269 | 1.781213351 | 0.016383797 |
| NR3C1 | 1.30339131 | 1.0482358 | 1.620655313 | 0.017136251 |
| UTS2 | 1.36030369 | 1.0518822 | 1.759157207 | 0.019000488 |
| CXCL12 | 1.06046229 | 1.0087833 | 1.114788761 | 0.021276951 |
| TNFRSF13C | 1.44836438 | 1.0563414 | 1.985872513 | 0.021429363 |
| ADIPOQ | 1.03665831 | 1.0053187 | 1.06897484 | 0.021524756 |
| SLC22A17 | 1.43319317 | 1.0534092 | 1.949900033 | 0.021951522 |
| SPP1 | 1.00273656 | 1.0003809 | 1.0050978 | 0.022769441 |
| CD19 | 1.25710993 | 1.0317422 | 1.531705606 | 0.023209452 |
| NOX4 | 1.6467724 | 1.0700107 | 2.534422537 | 0.023354821 |
| PLCG2 | 1.47899928 | 1.0502895 | 2.082700945 | 0.025032012 |
| SEMA3G | 1.10039444 | 1.0100189 | 1.198856675 | 0.02867267 |
| PAK3 | 250.411018 | 1.5938058 | 39343.36043 | 0.032304469 |
| MCHR2 | 496543807 | 5.0593118 | 4.87E+16 | 0.032954369 |
| CR2 | 1.18258634 | 1.0134971 | 1.379885991 | 0.033149766 |
| GRP | 1.14889937 | 1.0094037 | 1.307672849 | 0.035581092 |
| LTB4R | 1.06827404 | 1.0042476 | 1.136382535 | 0.036226424 |
| IL13 | 4.17E-06 | 3.74E-11 | 0.463693783 | 0.036654131 |
| LEP | 1.08616259 | 1.0028179 | 1.176434047 | 0.042453599 |
| MAPT | 2.26384496 | 1.0236145 | 5.006761731 | 0.04363314 |
| NR3C2 | 0.82989329 | 0.6923396 | 0.994776023 | 0.043736099 |
| IFNE | 1.84792653 | 1.0094177 | 3.382972791 | 0.046552803 |
| PLXNA1 | 1.09650691 | 1.0003909 | 1.201857656 | 0.049032048 |
| FAS | 0.87948775 | 0.7736954 | 0.999745775 | 0.04954722 |
| STC2 | 1.05568148 | 0.9990157 | 1.115561371 | 0.054231724 |
| OLR1 | 1.06741007 | 0.9978606 | 1.141806988 | 0.057740315 |
| PTGDS | 1.02570958 | 0.9987672 | 1.053378699 | 0.061603802 |
| HAMP | 2.63987546 | 0.9429719 | 7.390402728 | 0.06457779 |
| PLAU | 1.00767656 | 0.9994768 | 1.015943591 | 0.066591134 |
| MSTN | 0.00109237 | 7.37E-07 | 1.619384909 | 0.06716535 |
| CD79B | 1.10455877 | 0.992941 | 1.228723598 | 0.067305173 |
| SLIT2 | 1.35571132 | 0.9775179 | 1.880224575 | 0.068197349 |
| EDN3 | 0.86479436 | 0.7394585 | 1.011374197 | 0.069004983 |
| CD22 | 1.25988754 | 0.9821152 | 1.616222392 | 0.069071446 |
| KL | 1.1745821 | 0.9838399 | 1.402304515 | 0.075114314 |
| VEGFA | 1.03600722 | 0.9962798 | 1.077318806 | 0.076205556 |
| CRABP2 | 1.01408806 | 0.997986 | 1.030449955 | 0.086696154 |
| RETN | 1.26035302 | 0.9668582 | 1.642939671 | 0.087121714 |
| BMP5 | 0.55462218 | 0.2805332 | 1.096503848 | 0.090066262 |
| SEMA6A | 0.84372574 | 0.6920221 | 1.028685512 | 0.092898178 |
| GUCA2A | 0.98930873 | 0.9769644 | 1.001809073 | 0.093379733 |
| S100A2 | 1.00578691 | 0.9990161 | 1.012603602 | 0.094065827 |
| CTLA4 | 0.71153273 | 0.4750043 | 1.065840559 | 0.098800763 |
| CCL20 | 0.99345406 | 0.9856846 | 1.001284744 | 0.10111849 |
| CCL19 | 1.01940771 | 0.9961351 | 1.043223987 | 0.102821925 |
| NRG2 | 27.5352887 | 0.5091579 | 1489.109946 | 0.103433475 |
| NRG3 | 51046.2025 | 0.0864465 | 30142529685 | 0.109848943 |
| TG | 1.07932733 | 0.9806054 | 1.187988075 | 0.118810224 |
| CXCR5 | 37.8230307 | 0.3863474 | 3702.837427 | 0.120343131 |
| CXCL1 | 0.99516016 | 0.9890737 | 1.00128405 | 0.121142449 |
| GAST | 1.84785858 | 0.8492818 | 4.020551704 | 0.121600823 |
| TFR2 | 1.21647318 | 0.9481564 | 1.560720381 | 0.123256204 |
| BACH2 | 3.49378658 | 0.7116887 | 17.15152136 | 0.123316557 |
| VIP | 1.03112116 | 0.9910987 | 1.072759787 | 0.129192218 |
| ADRB2 | 2.11075459 | 0.7882902 | 5.65183375 | 0.137126228 |
| AHNAK | 1.01330712 | 0.995569 | 1.031361285 | 0.142345934 |
| LTB4R2 | 1.22449364 | 0.9298613 | 1.612482015 | 0.149261446 |
| TPM2 | 1.00502251 | 0.9982005 | 1.011891138 | 0.149395315 |
| SEMA3F | 1.03746346 | 0.9866982 | 1.090840598 | 0.150769076 |
| NRG4 | 4.042645 | 0.6003572 | 27.22209075 | 0.151116673 |
| STC1 | 1.04937243 | 0.9825303 | 1.120761844 | 0.151249548 |
| CMTM7 | 1.07010346 | 0.9751178 | 1.17434167 | 0.153099852 |
| PPY | 0.00298971 | 9.74E-07 | 9.17626472 | 0.155934879 |
| CSF2 | 1.15218879 | 0.9468331 | 1.402083377 | 0.157222987 |
| LGR6 | 0.95735017 | 0.9009949 | 1.017230373 | 0.159110557 |
| PYY | 0.85048047 | 0.6780788 | 1.066715243 | 0.161155882 |
| CSF1R | 1.03429694 | 0.9861792 | 1.084762483 | 0.165325006 |
| PDGFD | 1.23040388 | 0.9172966 | 1.650386345 | 0.166411906 |
| SLC11A1 | 1.1059487 | 0.9588228 | 1.275650186 | 0.166775311 |
| IL1RAP | 1.48929461 | 0.8437225 | 2.628824591 | 0.169493644 |
| SSTR2 | 7.33251226 | 0.4243527 | 126.7005909 | 0.170572432 |
| TNFRSF10C | 0.77102264 | 0.5314321 | 1.118630096 | 0.170830217 |
| GZMB | 0.98195616 | 0.9560597 | 1.008554074 | 0.181771026 |
| CCR9 | 4.9952902 | 0.4684841 | 53.263115 | 0.1828485 |
| CCL28 | 0.96342134 | 0.9114425 | 1.018364479 | 0.187880493 |
| SCTR | 5.26911962 | 0.4256414 | 65.22772059 | 0.195464961 |
| CTSS | 0.99181351 | 0.9795128 | 1.004268706 | 0.196708279 |
| PMCH | 0.04484078 | 0.0003932 | 5.114225072 | 0.198912921 |
| MMP9 | 1.00383342 | 0.9979625 | 1.009738899 | 0.201089992 |
| EDN2 | 0.66513442 | 0.3548083 | 1.246881159 | 0.20344804 |
| PTGS2 | 1.03435567 | 0.9818065 | 1.08971747 | 0.204170051 |
| NOD2 | 0.81409817 | 0.5920718 | 1.119384297 | 0.205566368 |
| MET | 1.01752574 | 0.9904918 | 1.04529751 | 0.206019616 |
| AZGP1 | 1.00699301 | 0.9961573 | 1.01794661 | 0.206781842 |
| TLR3 | 0.7832647 | 0.5355924 | 1.145467298 | 0.20779615 |
| RAET1E | 0.32681554 | 0.0568076 | 1.880179558 | 0.21030211 |
| PROCR | 0.98922718 | 0.9724392 | 1.006305001 | 0.214878948 |
| LCN1 | 1.26779719 | 0.8697315 | 1.848052826 | 0.217176143 |
| NOS1 | 3.404746 | 0.4833991 | 23.98079669 | 0.218653444 |
| PGLYRP4 | 0.1187426 | 0.0039316 | 3.58625128 | 0.220398094 |
| SCG2 | 1.05461578 | 0.9685085 | 1.148378613 | 0.221083238 |
| LCN12 | 1.06870569 | 0.9596685 | 1.19013169 | 0.226203069 |
| NR5A2 | 0.81661323 | 0.5873248 | 1.135414563 | 0.228302257 |
| BID | 0.96214637 | 0.9035038 | 1.024595201 | 0.229098034 |
| BMP6 | 1.13079802 | 0.925227 | 1.382043675 | 0.2298334 |
| CXCL3 | 0.98951401 | 0.9725275 | 1.006797232 | 0.23279742 |
| FGA | 0.47261258 | 0.1376936 | 1.622171149 | 0.233603323 |
| LEAP2 | 1.59572503 | 0.7393736 | 3.443912968 | 0.233789447 |
| SEMA6D | 0.77098828 | 0.497607 | 1.194562988 | 0.244350366 |
| MTNR1A | 1.46730642 | 0.7665028 | 2.8088457 | 0.247138192 |
| IL1RN | 0.96861655 | 0.9176566 | 1.022406412 | 0.247532248 |
| COLEC10 | 0.04828716 | 0.0002596 | 8.980356543 | 0.255673819 |
| AGT | 0.9832776 | 0.9550743 | 1.012313778 | 0.256069614 |
| RNASEL | 0.81050281 | 0.5638335 | 1.165086613 | 0.256487898 |
| TNFRSF12A | 1.00981794 | 0.9928923 | 1.027032155 | 0.257272509 |
| IL6R | 0.85723527 | 0.6559479 | 1.120290595 | 0.259271428 |
| SECTM1 | 0.97894358 | 0.9433378 | 1.015893266 | 0.260248726 |
| BTK | 1.220484 | 0.8579261 | 1.736258201 | 0.267905023 |
| DEFA6 | 0.99801065 | 0.9944913 | 1.001542411 | 0.269224785 |
| TNFSF9 | 0.97941916 | 0.9433954 | 1.016818497 | 0.276752515 |
| GNAI1 | 1.08597215 | 0.9356917 | 1.260389029 | 0.277790886 |
| A2M | 1.0045929 | 0.9962799 | 1.0129753 | 0.279761872 |
| MARCO | 1.03127177 | 0.9748349 | 1.090975949 | 0.283555052 |
| ILK | 1.10714773 | 0.915809 | 1.338462564 | 0.293045456 |
| TRIM27 | 1.04941651 | 0.9590376 | 1.148312624 | 0.293844444 |
| NPY | 2.05137421 | 0.535761 | 7.85450207 | 0.294215721 |
| HSP90AB1 | 1.00101412 | 0.9991181 | 1.002913731 | 0.294704783 |
| FLT3 | 0.12275907 | 0.0023473 | 6.420070807 | 0.29882775 |
| PRKCG | 0.8539396 | 0.6335249 | 1.15104045 | 0.299953965 |
| FGF16 | 8494.12329 | 0.0003075 | 2.35E+11 | 0.300715145 |
| CD1C | 0.77089019 | 0.4674341 | 1.271348616 | 0.308005726 |
| LGR4 | 0.97001154 | 0.9147676 | 1.028591743 | 0.308822156 |
| FAM19A5 | 1.16337587 | 0.8677375 | 1.559738271 | 0.311728551 |
| INPP5D | 1.03726589 | 0.9662523 | 1.113498566 | 0.311928579 |
| AREG | 0.99689403 | 0.9908357 | 1.002989403 | 0.317205803 |
| LIF | 1.02339401 | 0.977958 | 1.07094099 | 0.318268456 |
| APLN | 1.0434936 | 0.9596601 | 1.134650544 | 0.319083546 |
| IL1R2 | 0.96234005 | 0.8923249 | 1.037848831 | 0.319233768 |
| XCR1 | 0.37892993 | 0.0558763 | 2.569746316 | 0.320418501 |
| CXCL2 | 0.99074522 | 0.9727167 | 1.009107831 | 0.321039876 |
| CHGA | 0.96118798 | 0.8884304 | 1.039903966 | 0.324294815 |
| CNTFR | 1.0445927 | 0.9573843 | 1.139744958 | 0.32667002 |
| TNFRSF17 | 0.90788394 | 0.748302 | 1.101498179 | 0.327176369 |
| CXCL6 | 1.05138532 | 0.9510561 | 1.162298486 | 0.327449904 |
| FGFRL1 | 1.0047039 | 0.9952574 | 1.014240055 | 0.330228929 |
| MUC4 | 0.96361271 | 0.8943084 | 1.038287758 | 0.330392675 |
| LEPR | 1.47768497 | 0.6685673 | 3.2660179 | 0.334555127 |
| HTR3A | 0.21331066 | 0.0090353 | 5.035955854 | 0.338168985 |
| GH1 | 338.728547 | 0.0022294 | 51464384.32 | 0.338608695 |
| TNFRSF10B | 0.9806582 | 0.9420389 | 1.020860749 | 0.340696686 |
| RSAD2 | 0.93244903 | 0.8055046 | 1.079399385 | 0.348914492 |
| PLCG1 | 1.01968103 | 0.9788523 | 1.062212726 | 0.349898391 |
| CXCL13 | 0.97304818 | 0.9182575 | 1.031108154 | 0.355500929 |
| IL17B | 2.49289402 | 0.35735 | 17.39057259 | 0.356703762 |
| SH2D1B | 0.25196931 | 0.0133467 | 4.756862863 | 0.357801369 |
| FABP2 | 0.9628529 | 0.8878246 | 1.044221708 | 0.36043108 |
| AGRP | 5.01612334 | 0.148473 | 169.4684631 | 0.369218581 |
| ORM1 | 0.86459701 | 0.6287353 | 1.18893908 | 0.370697214 |
| S100P | 0.99903423 | 0.9969222 | 1.001150737 | 0.370865964 |
| ADCYAP1R1 | 3.65923257 | 0.2133634 | 62.75670375 | 0.370980982 |
| CD1A | 0.63675312 | 0.2348738 | 1.726265552 | 0.375057114 |
| HTR3C | 1.61516039 | 0.557758 | 4.677195674 | 0.376824104 |
| PF4 | 0.96920996 | 0.9041503 | 1.038951137 | 0.377702419 |
| FGFR4 | 0.99051236 | 0.969686 | 1.01178602 | 0.379262867 |
| S100A7 | 0.91081309 | 0.7392543 | 1.122185489 | 0.380307171 |
| SEMA3D | 1.27702242 | 0.7358099 | 2.216314565 | 0.384668723 |
| CST4 | 1.05356088 | 0.9356313 | 1.186354671 | 0.38898919 |
| CCR8 | 0.65677583 | 0.2521445 | 1.710743007 | 0.389397193 |
| ESM1 | 1.04808559 | 0.9409458 | 1.167424734 | 0.393315022 |
| EREG | 0.98892948 | 0.963934 | 1.014573107 | 0.394052518 |
| PNOC | 1.44811056 | 0.6165939 | 3.400981146 | 0.395349894 |
| IL17C | 0.77039771 | 0.4221174 | 1.406036954 | 0.395441995 |
| GLP2R | 0.37082574 | 0.0362095 | 3.797671072 | 0.403287966 |
| KIR2DL4 | 0.5792528 | 0.1585786 | 2.115882843 | 0.408760824 |
| PRL | 1.50097468 | 0.5659604 | 3.980711432 | 0.414448583 |
| INSL5 | 0.88326509 | 0.6537775 | 1.193306865 | 0.418714338 |
| CHP2 | 0.9888338 | 0.96227 | 1.016130917 | 0.418971232 |
| ELANE | 0.1645644 | 0.0020475 | 13.22676313 | 0.420113147 |
| HTR3B | 1.74E-11 | 7.86E-38 | 3.83E+15 | 0.423385105 |
| MIF | 0.99570285 | 0.985242 | 1.006274814 | 0.424198781 |
| CCL21 | 1.00655448 | 0.9905396 | 1.022828333 | 0.424658597 |
| IL16 | 1.18993757 | 0.7763467 | 1.823864746 | 0.424805282 |
| PTGER4 | 0.96484518 | 0.8836101 | 1.05354866 | 0.425154592 |
| S100A9 | 0.99822745 | 0.9938475 | 1.00262665 | 0.429085533 |
| ULBP3 | 1.12609764 | 0.8374704 | 1.514197939 | 0.431856506 |
| PSMD14 | 0.96311483 | 0.8756381 | 1.059330478 | 0.439175874 |
| FGF19 | 0.94969341 | 0.8319561 | 1.084092796 | 0.444673971 |
| LBP | 1.03763337 | 0.9437834 | 1.140815837 | 0.445005967 |
| FGF17 | 0.20952332 | 0.0036286 | 12.09842509 | 0.45010243 |
| PTN | 1.05104002 | 0.9236274 | 1.196028965 | 0.450243549 |
| PENK | 1.46692378 | 0.5412358 | 3.975836899 | 0.451327828 |
| PAK6 | 29.6300808 | 0.0038911 | 225630.9396 | 0.457409055 |
| REG1A | 0.99984705 | 0.9994424 | 1.000251848 | 0.458907221 |
| ROBO2 | 1.22011975 | 0.7143028 | 2.084119166 | 0.466426473 |
| GDF15 | 0.99855181 | 0.9946592 | 1.002459605 | 0.467080192 |
| FGF9 | 1.28581954 | 0.6519514 | 2.535974163 | 0.468162088 |
| IFITM1 | 0.99923575 | 0.9971733 | 1.001302474 | 0.468303308 |
| PGF | 1.07122386 | 0.8877195 | 1.292661249 | 0.472967631 |
| RLN2 | 0.74866793 | 0.3394458 | 1.651231573 | 0.473220288 |
| BMP4 | 0.99447705 | 0.9793535 | 1.009834126 | 0.478736467 |
| TEK | 1.09479046 | 0.8515283 | 1.40754698 | 0.479958898 |
| PCSK2 | 0.04298948 | 6.81E-06 | 271.3412116 | 0.48089987 |
| HSPA6 | 0.96687825 | 0.8791284 | 1.063386767 | 0.487757198 |
| TNFSF15 | 0.90734363 | 0.6881717 | 1.196318458 | 0.490644686 |
| SYTL1 | 1.0303509 | 0.9453823 | 1.122956214 | 0.49593516 |
| MMP12 | 0.99705595 | 0.9885857 | 1.005598789 | 0.498192115 |
| S100A3 | 0.92510022 | 0.7370352 | 1.161152692 | 0.501957876 |
| CXCL17 | 0.96557988 | 0.8712922 | 1.070070931 | 0.504055013 |
| CAMP | 0.48139888 | 0.0562463 | 4.120179104 | 0.504525228 |
| AR | 1.44576126 | 0.4823846 | 4.333110108 | 0.510386003 |
| DEFB1 | 1.01218949 | 0.9761799 | 1.049527355 | 0.512117525 |
| CCL4 | 0.97366796 | 0.8983922 | 1.055250982 | 0.515690687 |
| CD79A | 1.01021504 | 0.9797244 | 1.04165458 | 0.51571604 |
| TNFSF11 | 0.92160882 | 0.720366 | 1.179071176 | 0.516044666 |
| SPINK5 | 1.06079507 | 0.88759 | 1.267799553 | 0.516406754 |
| PTK2B | 0.96649157 | 0.8715541 | 1.071770418 | 0.518229414 |
| NR0B2 | 1.01989383 | 0.9606033 | 1.082843886 | 0.519166446 |
| PMP2 | 0.04472008 | 2.95E-06 | 677.5608767 | 0.526929832 |
| SSTR1 | 0.92382008 | 0.7226769 | 1.18094761 | 0.527087072 |
| PGR | 3.23948114 | 0.0844553 | 124.2579688 | 0.527584485 |
| CMKLR1 | 1.07241362 | 0.8630639 | 1.332544432 | 0.528085411 |
| IL17A | 0.70315589 | 0.234607 | 2.107474197 | 0.529455739 |
| S100A5 | 0.85165746 | 0.5160881 | 1.405419758 | 0.529816147 |
| NDRG1 | 1.00336206 | 0.9928192 | 1.0140169 | 0.533433304 |
| PGC | 0.98147103 | 0.925368 | 1.040975495 | 0.533436895 |
| PI15 | 1.09753512 | 0.8160936 | 1.476035748 | 0.538136977 |
| ANGPTL3 | 65.0743819 | 0.0001047 | 40442850.63 | 0.539551712 |
| IL5RA | 9.14367074 | 0.0076622 | 10911.62487 | 0.540370217 |
| CXCL5 | 1.00214673 | 0.9952243 | 1.009117299 | 0.544278331 |
| NCR2 | 7.08827462 | 0.0124141 | 4047.318625 | 0.545355071 |
| LIFR | 1.36487418 | 0.497661 | 3.743274333 | 0.545648511 |
| PTX3 | 1.15915482 | 0.710572 | 1.890927256 | 0.554180935 |
| INHBE | 1.48358194 | 0.3967291 | 5.547905002 | 0.557765947 |
| CCL11 | 0.97798997 | 0.9061551 | 1.055519457 | 0.567468895 |
| ACVR2B | 1.20917236 | 0.6301288 | 2.320315642 | 0.567886337 |
| PTGDR | 0.95490438 | 0.8142341 | 1.119877392 | 0.570365762 |
| IL10RA | 1.03743778 | 0.9136546 | 1.177991269 | 0.570738932 |
| FAM19A4 | 0.05685436 | 2.80E-06 | 1155.200804 | 0.57102296 |
| TNFRSF9 | 0.80441835 | 0.3775807 | 1.713776232 | 0.572767302 |
| OPRD1 | 0.7529114 | 0.2802511 | 2.022741938 | 0.573530273 |
| ACVR1C | 0.83870499 | 0.4509384 | 1.559916217 | 0.57850112 |
| CD70 | 0.9631251 | 0.8414881 | 1.102344705 | 0.585455685 |
| IL33 | 1.00486057 | 0.987485 | 1.022541912 | 0.585866709 |
| BIRC5 | 0.98963923 | 0.9532277 | 1.027441568 | 0.58607525 |
| RXRG | 0.17639293 | 0.0003157 | 98.55125909 | 0.59085694 |
| RAC3 | 1.02332251 | 0.9394062 | 1.114734957 | 0.597420984 |
| LTBP4 | 1.0080342 | 0.9781504 | 1.038830941 | 0.602253106 |
| CCR7 | 0.92710938 | 0.6968463 | 1.233459739 | 0.603371503 |
| TNFRSF10A | 0.97551515 | 0.8873359 | 1.07245719 | 0.608069249 |
| IL1B | 0.99371224 | 0.9696993 | 1.018319825 | 0.613284345 |
| GREM2 | 0.94290748 | 0.7497477 | 1.185831606 | 0.61521816 |
| BMP3 | 0.86721686 | 0.4958786 | 1.516631477 | 0.617390461 |
| NR1H4 | 0.8805696 | 0.531107 | 1.459974685 | 0.62198867 |
| CMA1 | 0.87340752 | 0.5057028 | 1.508476185 | 0.627342318 |
| IL17F | 0.639824 | 0.1052539 | 3.889402037 | 0.627712032 |
| IL2 | 6.68269423 | 0.0028722 | 15548.46734 | 0.631049544 |
| ZC3HAV1L | 0.96094284 | 0.8157937 | 1.131917481 | 0.633469697 |
| ULBP1 | 1.24995962 | 0.4995103 | 3.12786172 | 0.633542781 |
| CTSG | 0.94135553 | 0.7319401 | 1.2106868 | 0.637824708 |
| IL23A | 0.98412787 | 0.9192811 | 1.05354899 | 0.645485319 |
| PDIA2 | 1.01298682 | 0.9583287 | 1.070762404 | 0.648434474 |
| TNFSF10 | 0.99261754 | 0.9612338 | 1.02502592 | 0.651240079 |
| PLXNA2 | 1.03502172 | 0.8828462 | 1.213427655 | 0.67138533 |
| ACVRL1 | 0.98998894 | 0.9440493 | 1.038164059 | 0.678121387 |
| LCN6 | 0.82454347 | 0.3309716 | 2.054169851 | 0.678690523 |
| SLC10A2 | 1.84129252 | 0.1014258 | 33.42699502 | 0.679795974 |
| IFNG | 0.86916735 | 0.4462073 | 1.693051352 | 0.680203644 |
| CYSLTR1 | 0.7320727 | 0.1654951 | 3.238347186 | 0.681006987 |
| BMP2 | 0.98119577 | 0.8951925 | 1.075461598 | 0.685040247 |
| AQP9 | 0.98079925 | 0.89285 | 1.077411844 | 0.685873981 |
| CSF2RB | 0.9746424 | 0.858877 | 1.106011509 | 0.690537524 |
| CCR10 | 0.87162394 | 0.4404541 | 1.724875206 | 0.693182894 |
| NMB | 1.01217729 | 0.9514041 | 1.07683248 | 0.701630025 |
| CX3CR1 | 0.75284902 | 0.1730662 | 3.27494205 | 0.705085409 |
| KNG1 | 0.83524246 | 0.327005 | 2.13339228 | 0.706706085 |
| MASP1 | 0.89172137 | 0.4893978 | 1.624786622 | 0.708127637 |
| CCL26 | 1.0246544 | 0.8985964 | 1.168396265 | 0.716137025 |
| CD40LG | 0.86894213 | 0.4068607 | 1.855820354 | 0.716716268 |
| RBP4 | 1.00196106 | 0.9914027 | 1.012631816 | 0.717001342 |
| NDP | 0.92564068 | 0.6077199 | 1.409877578 | 0.718906073 |
| ULBP2 | 0.9730999 | 0.8382986 | 1.129577719 | 0.720026619 |
| NPPC | 1.41553282 | 0.2070654 | 9.676812635 | 0.72309286 |
| IL13RA2 | 0.9413303 | 0.673058 | 1.316532613 | 0.723901999 |
| HMOX1 | 0.99187365 | 0.9478472 | 1.037945092 | 0.724660464 |
| CD48 | 0.97272786 | 0.8334839 | 1.13523424 | 0.725739569 |
| CCL8 | 1.03243341 | 0.8637858 | 1.234008138 | 0.725762034 |
| IL11 | 1.02168088 | 0.9052594 | 1.153074864 | 0.728227816 |
| MPO | 1.25148064 | 0.3527666 | 4.439773561 | 0.728427716 |
| IL1A | 1.02801691 | 0.8788205 | 1.20254229 | 0.729812403 |
| CD1D | 1.06834311 | 0.7340432 | 1.554890862 | 0.729906287 |
| TUBB3 | 0.91504007 | 0.5386293 | 1.554498363 | 0.742626712 |
| OBP2A | 1.05242012 | 0.7734231 | 1.432059779 | 0.745101756 |
| DES | 0.99962909 | 0.9973226 | 1.001940884 | 0.752937011 |
| CALCA | 0.9953078 | 0.9665112 | 1.024962411 | 0.753536578 |
| FGF18 | 0.95489542 | 0.7030789 | 1.296903242 | 0.767619741 |
| SSTR5 | 0.97360015 | 0.8146773 | 1.163524842 | 0.768566336 |
| PIK3CG | 1.11631337 | 0.5337248 | 2.334827692 | 0.770090452 |
| OXT | 1.2312379 | 0.2951776 | 5.135710006 | 0.77528184 |
| ANGPTL7 | 1.34729106 | 0.1638714 | 11.07694022 | 0.781531489 |
| R3HDML | 1.02102611 | 0.8776668 | 1.187801874 | 0.787500217 |
| AVPR1B | 20.6196117 | 3.44E-09 | 1.24E+11 | 0.792203568 |
| CCL23 | 0.92246287 | 0.4948005 | 1.719759397 | 0.79953231 |
| GDNF | 0.82225675 | 0.1731168 | 3.905492409 | 0.805542751 |
| CRABP1 | 0.95449865 | 0.6566827 | 1.387378907 | 0.807186822 |
| CCL13 | 1.00736969 | 0.9496765 | 1.068567745 | 0.807216516 |
| CSRP1 | 1.00274764 | 0.9808615 | 1.025122087 | 0.807464955 |
| EDNRA | 1.0093887 | 0.9360276 | 1.088499449 | 0.8082102 |
| HSPA2 | 0.99619674 | 0.9659273 | 1.027414799 | 0.808748912 |
| VIPR2 | 0.79078475 | 0.115162 | 5.430096592 | 0.811273306 |
| RFXAP | 0.97904875 | 0.8219344 | 1.166195859 | 0.812462938 |
| FGF10 | 1.14944939 | 0.3586891 | 3.683507731 | 0.814666719 |
| THRB | 1.04595913 | 0.714405 | 1.531387025 | 0.817308175 |
| LGR5 | 0.99787244 | 0.9799568 | 1.016115583 | 0.817770301 |
| UCN3 | 0.97937671 | 0.8190637 | 1.171067285 | 0.819265677 |
| LCN10 | 1.41413792 | 0.0716034 | 27.92866146 | 0.819903592 |
| CCL5 | 0.99796891 | 0.9802998 | 1.015956463 | 0.823478412 |
| CSF3 | 0.98972449 | 0.9022997 | 1.085619977 | 0.82672822 |
| ADRM1 | 0.99903661 | 0.990127 | 1.008026419 | 0.832979982 |
| NR5A1 | 0.89400382 | 0.3154579 | 2.533596179 | 0.833030277 |
| CDK4 | 0.99740615 | 0.9727533 | 1.022683788 | 0.83882672 |
| FGF8 | 0.95668341 | 0.6227477 | 1.469685279 | 0.839793387 |
| CYSLTR2 | 1.30890746 | 0.0959408 | 17.85724641 | 0.839994979 |
| S100A11 | 1.00011544 | 0.9989795 | 1.001252715 | 0.842209699 |
| CXCL16 | 1.00158944 | 0.9857707 | 1.017662026 | 0.844978424 |
| OSM | 1.00512557 | 0.9544398 | 1.058503043 | 0.846446915 |
| GPR17 | 1.12199123 | 0.333192 | 3.778194957 | 0.852592454 |
| CMTM5 | 0.29868167 | 3.16E-07 | 282146.0172 | 0.863328766 |
| OBP2B | 1.00408545 | 0.9585164 | 1.051820945 | 0.863397591 |
| AEN | 1.00937744 | 0.9067551 | 1.123614112 | 0.864518838 |
| FAM3D | 1.00020771 | 0.9978104 | 1.0026108 | 0.865304496 |
| INHBB | 1.0045335 | 0.9515045 | 1.060517942 | 0.8701518 |
| NCR3 | 0.93141859 | 0.3958229 | 2.191739174 | 0.870736834 |
| FGFR2 | 1.01257352 | 0.87051 | 1.177821207 | 0.871304476 |
| MUC5AC | 0.99766402 | 0.9694915 | 1.026655157 | 0.872863672 |
| CCL16 | 0.58684455 | 0.0005526 | 623.2078134 | 0.88082459 |
| ANGPT1 | 0.93186069 | 0.3530881 | 2.459341817 | 0.886663014 |
| VIPR1 | 1.00736039 | 0.9076632 | 1.118008299 | 0.890304482 |
| GDF5 | 1.11267207 | 0.2417369 | 5.121433196 | 0.890978718 |
| S100B | 1.01191539 | 0.8428065 | 1.21495587 | 0.89897454 |
| WNT5A | 1.00850791 | 0.8810566 | 1.154395945 | 0.90218502 |
| TNFRSF13B | 1.10627895 | 0.2178723 | 5.617295725 | 0.903031131 |
| CD209 | 0.98540789 | 0.7778034 | 1.248424307 | 0.903073944 |
| NODAL | 0.94535774 | 0.3707927 | 2.410245295 | 0.906325518 |
| CCL24 | 1.00023389 | 0.9963138 | 1.00416944 | 0.907078316 |
| EBI3 | 1.01369528 | 0.7667134 | 1.340237637 | 0.923939737 |
| PAEP | 0.99885276 | 0.9742508 | 1.02407592 | 0.928116019 |
| OASL | 1.00265819 | 0.9455787 | 1.063183282 | 0.929264992 |
| NRG1 | 0.94877853 | 0.2961561 | 3.039548332 | 0.929468975 |
| LCN2 | 1.00001465 | 0.9996875 | 1.000341876 | 0.930051823 |
| AGTR1 | 1.03715411 | 0.4550784 | 2.363743459 | 0.930832773 |
| VGF | 0.99789524 | 0.9490258 | 1.049281222 | 0.934453734 |
| LMBR1 | 1.00741241 | 0.8309191 | 1.22139421 | 0.940095371 |
| CMTM8 | 0.99768089 | 0.9381815 | 1.060953742 | 0.941005459 |
| PRKCB | 0.97785665 | 0.5358063 | 1.784607005 | 0.941843429 |
| TSHB | 0.71903637 | 9.98E-05 | 5179.258049 | 0.941978485 |
| TLR7 | 0.96563888 | 0.3737201 | 2.495071675 | 0.942448947 |
| SST | 0.99724666 | 0.9222728 | 1.078315329 | 0.94487706 |
| IL20RA | 1.00334359 | 0.9075442 | 1.109255446 | 0.948018738 |
| EDNRB | 0.99170655 | 0.7634678 | 1.288177236 | 0.950239626 |
| AGTR2 | 0.63535245 | 3.12E-07 | 1292823.115 | 0.951199629 |
| NFAT5 | 1.00701976 | 0.7995928 | 1.268256569 | 0.952599275 |
| CLEC4M | 0.59918441 | 8.18E-09 | 43866372.1 | 0.955791822 |
| FGF20 | 0.99412692 | 0.8066504 | 1.225175595 | 0.95594266 |
| ANGPTL1 | 0.99155631 | 0.7306803 | 1.345573335 | 0.956587027 |
| AMELX | 0.98324854 | 0.5105875 | 1.893461453 | 0.959702415 |
| TACR1 | 0.95319018 | 0.134014 | 6.779677778 | 0.961800524 |
| GCG | 0.99979384 | 0.9902771 | 1.009402028 | 0.966297783 |
| TNFSF4 | 0.9941987 | 0.7373789 | 1.34046564 | 0.969560397 |
| SERPIND1 | 0.99633762 | 0.8218427 | 1.207881599 | 0.970205749 |
| F2R | 1.00092747 | 0.9511514 | 1.053308401 | 0.971584866 |
| TNFRSF11B | 0.99960108 | 0.9760568 | 1.023713242 | 0.973826707 |
| CXCL11 | 1.00033402 | 0.9802967 | 1.020780909 | 0.974193337 |
| PPBP | 0.99999547 | 0.9996635 | 1.000327552 | 0.978641621 |
| PGLYRP3 | 1.01047336 | 0.346473 | 2.947001145 | 0.984778763 |
| BMP7 | 0.99971702 | 0.9703173 | 1.030007566 | 0.985173384 |
| UCN2 | 1.0043037 | 0.4813531 | 2.095396954 | 0.990868644 |
| INHA | 1.00059224 | 0.8692037 | 1.15184143 | 0.993422728 |
| GHR | 0.99927651 | 0.6597429 | 1.513549545 | 0.997273901 |
| OGN | 0.99995711 | 0.8952494 | 1.116911373 | 0.999393573 |
