# Supplementary material for: Construction of an immune-related signature with prognostic value for colon cancer
Source: PeerJ. 2021 May 5;9:e10812. doi: 10.7717/peerj.10812 (PMC8106397; doi:10.7717/peerj.10812)
Supplement: Table S5 — Using Mann Whitney U test and Kruskal-Wallis test, we compared expression levels of every gene in this immune-related signature in different clinical subgroups. If P < 0.05, this gene was considered to be related to this clinical phenotype. As shown in this table, CD1B, PLCG2, DKK1 and GRP had strong correlation with tumor stage, TNM classification or vital status of patients. [file peerj-09-10812-s007.docx]

| **Table S5 Validation of correlation between risk scores and clinical phenotypes.** | | | | | | | | | |
| --- | --- | --- | --- | --- | --- | --- | --- | --- | --- |
| **Id** | **fustat** | **T** | **N** | **M** | **stage** | **age** | **gender** | **lymphatic_invasion** | **venous_invasion** |
| CD1B | 3.00E-05 | 1.31E-05 | 0.001 | 5.09E-05 | 8.38E-05 | 0.671 | 0.043 | 0.196 | 0.221 |
| LTB4R | 0.005 | 0.201 | 0.423 | 0.243 | 0.656 | 0.791 | 0.803 | 0.203 | 0.348 |
| IL13 | 0.297 | 0.074 | 0.253 | 8.91E-04 | 0.01 | 0.259 | 0.134 | 0.57 | 0.517 |
| PLCG2 | 0.532 | 0.597 | 0.346 | 0.828 | 0.785 | 0.351 | 0.189 | 0.504 | 0.182 |
| BDNF | 0.059 | 0.699 | 0.036 | 0.446 | 0.207 | 0.022 | 0.804 | 0.943 | 0.114 |
| DKK1 | 0.724 | 0.173 | 0.006 | 0.021 | 0.03 | 0.01 | 0.32 | 0.077 | 0.047 |
| GRP | 0.684 | 6.90E-04 | 1.33E-05 | 0.002 | 4.65E-06 | 0.053 | 0.651 | 1.42E-04 | 0.031 |
| IGF1 | 0.337 | 0.804 | 0.148 | 0.629 | 0.518 | 0.989 | 0.291 | 0.243 | 0.331 |
| SPP1 | 0.777 | 0.021 | 0.1 | 0.397 | 0.04 | 0.595 | 0.081 | 0.609 | 0.285 |
| UCN | 0.002 | 0.102 | 0.29 | 0.097 | 0.253 | 0.009 | 0.145 | 0.3 | 0.764 |
| UTS2 | 0.193 | 0.37 | 0.733 | 0.431 | 0.687 | 0.332 | 0.876 | 0.85 | 0.459 |
| FAS | 0.027 | 0.368 | 0.002 | 2.64E-05 | 3.44E-05 | 0.043 | 0.562 | 0.018 | 0.421 |
| riskScore | 3.20E-11 | 1.08E-04 | 5.80E-08 | 7.87E-08 | 8.34E-09 | 0.416 | 0.092 | 0.026 | 0.062 |
