# Supplementary material for: Construction of an immune-related signature with prognostic value for colon cancer
Source: PeerJ. 2021 May 5;9:e10812. doi: 10.7717/peerj.10812 (PMC8106397; doi:10.7717/peerj.10812)
Supplement: Table S6 — We exerted Wilcoxon signed-ranked tests to screen differentially expressed transcription factors (—FC (Fold change) —> 1, P < 0.05 and FDR < 0.25) between normal tissue samples and primary tumor tissue samples from TCGA. FDR, false discovery rate; FC, fold change; TCGA, The Cancer Genome Atlas. [file peerj-09-10812-s008.docx]

| Table S6 Screening of differentially expressed TFs. | | | | | |
| --- | --- | --- | --- | --- | --- |
| ID | **conMean** | **treatMean** | **logFC** | **pValue** | **FDR** |
| RXRG | 0.7206077 | 0.0512368 | -3.813963 | 3.46E-26 | 8.54E-24 |
| SPIB | 13.82464 | 0.621519 | -4.4753 | 8.16E-26 | 1.42E-23 |
| CBX2 | 0.2372761 | 3.144468 | 3.7281771 | 4.06E-25 | 3.85E-23 |
| SALL4 | 0.0337644 | 1.057694 | 4.9692754 | 4.71E-25 | 4.18E-23 |
| TEAD4 | 2.714068 | 14.17206 | 2.3845202 | 7.78E-25 | 5.62E-23 |
| TCF21 | 3.793402 | 0.846376 | -2.164122 | 8.32E-25 | 5.82E-23 |
| CBX8 | 1.187052 | 4.229466 | 1.8330927 | 1.42E-24 | 8.86E-23 |
| PDX1 | 0.2865654 | 8.182502 | 4.8356057 | 2.31E-24 | 1.27E-22 |
| NR5A2 | 6.82378 | 1.487008 | -2.198158 | 3.13E-24 | 1.59E-22 |
| MXI1 | 25.8699 | 9.314516 | -1.473722 | 4.89E-24 | 2.24E-22 |
| KLF4 | 111.9147 | 21.90414 | -2.353124 | 5.29E-24 | 2.38E-22 |
| SOX9 | 17.41105 | 69.42267 | 1.9954039 | 8.00E-24 | 3.24E-22 |
| SOX4 | 8.616198 | 30.75839 | 1.8358565 | 2.19E-23 | 6.55E-22 |
| CBX7 | 7.696595 | 2.472874 | -1.638032 | 4.06E-23 | 1.06E-21 |
| EZH2 | 3.290098 | 8.953755 | 1.4443621 | 4.63E-23 | 1.18E-21 |
| KAT2B | 6.092363 | 2.257267 | -1.432425 | 5.46E-23 | 1.34E-21 |
| E2F1 | 3.783185 | 14.73296 | 1.9613743 | 1.07E-22 | 2.35E-21 |
| CDK2 | 5.0672 | 11.53158 | 1.1863295 | 1.23E-22 | 2.65E-21 |
| MYC | 25.65659 | 104.3415 | 2.023912 | 1.26E-22 | 2.70E-21 |
| CBFB | 6.082557 | 17.69097 | 1.5402631 | 1.44E-22 | 3.01E-21 |
| TAT | 0.4367701 | 0.0355824 | -3.617641 | 2.24E-22 | 4.46E-21 |
| E2F7 | 0.3631703 | 1.543279 | 2.0872804 | 4.13E-22 | 7.49E-21 |
| RUNX1 | 1.907061 | 5.557489 | 1.5430821 | 4.64E-22 | 8.17E-21 |
| CENPA | 2.116262 | 6.499204 | 1.6187445 | 1.08E-21 | 1.63E-20 |
| TRIM28 | 55.07822 | 113.1241 | 1.0383526 | 1.43E-21 | 2.10E-20 |
| MYBL2 | 9.84128 | 44.67662 | 2.1826022 | 4.32E-21 | 5.54E-20 |
| EPAS1 | 42.22793 | 19.6364 | -1.104667 | 6.83E-21 | 8.33E-20 |
| TCF7 | 1.421091 | 5.800538 | 2.0291883 | 1.59E-20 | 1.77E-19 |
| MYH11 | 282.6829 | 15.78259 | -4.162779 | 2.00E-20 | 2.17E-19 |
| CEBPB | 11.35183 | 33.16449 | 1.5467147 | 2.31E-20 | 2.47E-19 |
| CBX3 | 21.99767 | 55.07583 | 1.3240688 | 4.01E-20 | 4.02E-19 |
| E2F3 | 2.929829 | 6.219021 | 1.0858711 | 5.12E-20 | 5.02E-19 |
| NR3C1 | 4.742841 | 1.787216 | -1.408037 | 5.44E-20 | 5.30E-19 |
| BRCA1 | 1.317856 | 3.603657 | 1.4512691 | 1.03E-19 | 9.42E-19 |
| FOXM1 | 5.195551 | 14.32844 | 1.4635331 | 1.63E-19 | 1.44E-18 |
| LEF1 | 0.6533124 | 3.154785 | 2.2716969 | 1.72E-19 | 1.50E-18 |
| ARID3A | 1.53941 | 7.324995 | 2.2504507 | 1.82E-19 | 1.59E-18 |
| BHLHE40 | 19.01388 | 52.57605 | 1.4673531 | 2.00E-19 | 1.72E-18 |
| FOSL1 | 1.555937 | 9.959589 | 2.6783027 | 2.77E-19 | 2.33E-18 |
| MEF2C | 2.856687 | 1.163163 | -1.296289 | 8.00E-19 | 6.12E-18 |
| ASCL1 | 0.0788812 | 0.029792 | -1.404755 | 1.15E-18 | 8.52E-18 |
| LMO2 | 3.957064 | 1.877993 | -1.075239 | 2.98E-18 | 2.04E-17 |
| TCF7L1 | 3.265843 | 1.267702 | -1.365239 | 3.19E-18 | 2.17E-17 |
| NCAPG | 2.01249 | 5.357965 | 1.4127036 | 3.45E-18 | 2.33E-17 |
| PRKDC | 7.693075 | 19.83464 | 1.3663899 | 6.26E-18 | 4.04E-17 |
| IRF4 | 4.361194 | 0.9400894 | -2.213853 | 6.69E-18 | 4.29E-17 |
| POLR3G | 0.4550346 | 1.625365 | 1.8367154 | 8.34E-18 | 5.25E-17 |
| MAF | 8.4874 | 2.976325 | -1.51179 | 9.81E-18 | 6.14E-17 |
| DNMT1 | 5.750318 | 11.89435 | 1.0485623 | 1.15E-17 | 7.15E-17 |
| FLI1 | 2.277616 | 1.083357 | -1.072016 | 3.71E-17 | 2.12E-16 |
| TFAP2A | 0.0783337 | 0.9737948 | 3.6359124 | 5.98E-17 | 3.31E-16 |
| CHD7 | 1.743749 | 4.149036 | 1.2505833 | 6.81E-17 | 3.72E-16 |
| PBX1 | 4.506581 | 2.062877 | -1.127376 | 2.99E-16 | 1.49E-15 |
| FOXA2 | 6.092471 | 18.66352 | 1.615122 | 3.01E-16 | 1.50E-15 |
| FOXP2 | 1.243261 | 0.3484469 | -1.835119 | 6.21E-16 | 2.97E-15 |
| IKZF1 | 2.458131 | 1.031449 | -1.252889 | 2.26E-15 | 1.01E-14 |
| H2AFX | 24.55562 | 54.72914 | 1.1562557 | 9.29E-15 | 3.83E-14 |
| MEIS1 | 2.270937 | 0.826079 | -1.458936 | 2.45E-14 | 9.55E-14 |
| TP73 | 0.1609685 | 0.8597401 | 2.417122 | 3.29E-14 | 1.26E-13 |
| EBF1 | 1.185617 | 0.5415055 | -1.13059 | 4.46E-14 | 1.69E-13 |
| OGT | 9.129014 | 20.46986 | 1.1649702 | 4.81E-14 | 1.82E-13 |
| AR | 0.426494 | 0.1651362 | -1.368869 | 1.31E-12 | 4.22E-12 |
| FOXP3 | 0.561577 | 1.635749 | 1.5423959 | 1.56E-11 | 4.47E-11 |
| BACH2 | 0.414281 | 0.1947672 | -1.088859 | 5.82E-11 | 1.58E-10 |
| ELF5 | 0.0029369 | 0.8279609 | 8.1391017 | 1.20E-10 | 3.15E-10 |
| PAX5 | 1.110563 | 0.3574144 | -1.635622 | 1.97E-09 | 4.59E-09 |
| HOXA9 | 5.215552 | 12.00611 | 1.2028765 | 2.53E-09 | 5.82E-09 |
| GREB1 | 0.0623724 | 0.1505501 | 1.2712648 | 2.40E-07 | 4.60E-07 |
| HOXC11 | 0.0347509 | 0.5903364 | 4.0864144 | 9.36E-07 | 1.70E-06 |
| SCML2 | 0.479723 | 1.044082 | 1.1219614 | 2.55E-05 | 4.08E-05 |
| BATF | 2.481434 | 5.637701 | 1.1839331 | 0.0001956 | 0.0002889 |
| TFAP2C | 0.6800372 | 1.438232 | 1.0806103 | 0.000283 | 0.0004129 |
| NFE2 | 0.156996 | 0.5623198 | 1.8406634 | 0.0003379 | 0.0004901 |
| EPO | 0.0147161 | 0.0393893 | 1.4204052 | 0.0216487 | 0.026295 |
